# Comparative study of palmitoleic acid, sea buckthorn oil, and lovastatin in hepatocellular steatosis model

**DOI:** 10.1038/s41598-026-37006-y

**Published:** 2026-01-24

**Authors:** Marcin Szustak, Marta Pichlak, Eliza Korkus, Daria Kamińska, Maria Koziołkiewicz, Grzegorz Dąbrowski, Sylwester Czaplicki, Iwona Konopka, Edyta Gendaszewska-Darmach

**Affiliations:** 1 Faculty of Biotechnology and Food Sciences, Institute of Molecular and Industrial Biotechnology, Lodz University of Technology, Lodz, 90-537 Poland; 2 Faculty of Food Sciences, Chair of Plant Food Chemistry and Processing, University of Warmia and Mazury in Olsztyn, Olsztyn, 10-957 Poland

**Keywords:** Cholesterol, HMG-CoA reductase, Liver steatosis, Palmitoleic acid isomers, Prenylation, Sea buckthorn oil, Cell biology, Diseases, Molecular medicine

## Abstract

Palmitoleic acid (POA) has been associated with various metabolic effects, including regulating lipid metabolism and cholesterol production. In this study, we explored the impact of both *cis*- (cPOA) and *trans*-palmitoleic acid (tPOA), along with sea buckthorn oil (SBO), a natural source rich in cPOA, on lipid content, metabolic activity, and Rap1 prenylation in HepG2 cells. We employed a free fatty acids-induced steatosis model. Our findings revealed that neither cPOA, tPOA, SBO, nor digested SBO impaired the metabolic activity of HepG2 cells, even under steatotic conditions. All treatments led to triacylglycerol (TAG) accumulation, with tPOA demonstrating the beneficial effect. While cholesterol levels remained unchanged in normal cells, a reduction was noted in steatotic cells, comparable to the effect of lovastatin. Molecular docking suggested that both POA isomers may bind and block the catalytic pocket of 3-hydroxy-3-methylglutaryl coenzyme A reductase (HMGCR). To further explore this, we examined their influence on Rap1 prenylation and subcellular localization. Under steatotic conditions, both POA isomers, particularly at concentrations of 50 and 100 µM, elevated cytosolic Rap1 levels, whereas no significant changes were observed in membrane or cytosolic Rap1a protein levels in normal HepG2 cultures. Notably, unlike lovastatin, POA isomers and digested SBO enhanced glucose-stimulated insulin secretion from pancreatic cells. Our results highlight the potential of POA isomers and SBO as modulators of lipid metabolism, cholesterol production, and insulin secretion, suggesting their relevance in managing metabolic disorders.

**Supplementary Information:**

The online version contains supplementary material available at 10.1038/s41598-026-37006-y.

## Introduction

Metabolic dysfunction-associated steatotic liver disease (MASLD), formerly known as nonalcoholic fatty liver disease (NAFLD), is a prevalent chronic liver disorder affecting over 30% of the global population^1^. MASLD is characterized by the presence of hepatic steatosis in individuals with at least one metabolic risk factor, including obesity, diabetes mellitus, dyslipidemia, or hypertension. The disease progresses from a healthy liver to steatosis (fat accumulation), followed by inflammation, fibrosis, and ultimately, cirrhosis. The clinical spectrum ranges from the relatively benign Metabolic Dysfunction-Associated Steatotic Liver (MASL) to the more severe Metabolic Dysfunction-Associated Steatohepatitis (MASH; previously referred to as Nonalcoholic Steatohepatitis, NASH)^2^. The accumulation of triacylglycerols (TAGs) in the liver is frequently associated with a high-fat diet (HFD), which upregulates the mRNA and protein expression of CD36, a fatty acid translocase, thereby exacerbating hepatic steatosis. Due to its strong association with obesity, type 2 diabetes, hyperlipidemia, and other components of the metabolic syndrome, MASLD has escalated to pandemic proportions, posing a major public health concern^3^. Lipidomic studies have also revealed an accumulation of free cholesterol in the liver in MASLD cases^4^.

Palmitoleic acid (POA, 16:1n-7), a monounsaturated fatty acid classified as a lipokine, has demonstrated promising nutraceutical potential, particularly in the management of MASLD^5^. Palmitoleate exists in two natural isomeric forms: *cis* (cPOA) and *trans* (tPOA). These isomers differ not only in their geometric configuration but also in their biosynthetic origins, dietary sources, and biological effects. Endogenous cPOA is synthesized primarily in the liver, and to a lesser extent in the adipose tissue, through the desaturation of palmitic acid. TAGs in subcutaneous adipose tissue comprise approximately 7.2 mol% of cPOA, making it the second most abundant monounsaturated fatty acid after oleic acid^6^. Sea buckthorn fruit oil (SBO) is the richest known natural source of cPOA, containing between 16% and 54% of this fatty acid. Sea buckthorn, a thorny shrub cultivated in Northwestern Europe and Central Asia, is unique in its cPOA content and is remarkably nutrient-dense, comprising over 200 bioactive compounds. Its oil contains eight types of saturated fatty acids and eleven types of unsaturated fatty acids, including the essential α-linolenic acid (ALA) and linoleic acid (LA)^7,8^In turn, tPOA is predominantly found in *trans* fats derived from ruminant sources, such as dairy and meat products. Its concentration in dairy fat is relatively low, accounting for 0.05%, 0.04%, and 0.08% of total fatty acids in cow, ewe, and goat milk, respectively^9^. Recent findings have shown that in regions where partially hydrogenated oils have been discontinued, ruminant-delivered fats are the sole contributors to circulating tPOA in humans^10^. Moreover, plasma levels of tPOA, which range from 0.02% to 0.55% of total fatty acids, are primarily influenced by dietary intake^11^.

Experimental studies in high-fat diet (HFD)-fed db/db mice have revealed that cPOA and tPOA exert distinct and sometimes opposing effects on lipid metabolism and liver pathology^12^. Administration of tPOA ameliorated hypercholesterolemia by reducing serum levels of total cholesterol, low-density lipoprotein (LDL), high-density lipoprotein (HDL), hepatic free cholesterol, and total bile acids (TBAs). In contrast, cPOA did not significantly influence these parameters, except for a reduction in TBAs. Despite this, histological analysis revealed that cPOA was more effective than tPOA in alleviating hepatic lipidosis. These effects are mediated through differential regulation of key genes involved in cholesterol metabolism: tPOA downregulated the expression of 3-hydroxy-3-methylglutaryl coenzyme A (HMG-CoA) reductase (HMGCR) and liver X receptor alpha (LXRα), while upregulating cholesterol 7-alpha hydroxylase (CYP7A1) in the liver. Conversely, cPOA decreased the expression of both HMGCR and CYP7A1, suggesting that tPOA and cPOA modulate cholesterol metabolism via distinct mechanisms. In vitro studies using HepG2 cell model further support the lipid-lowering potential of tPOA, which significantly reduces lipid accumulation compared to palmitic acid (PA) and mitigates PA-induced steatosis^12^. However, the effects of cPOA in this cellular context remain underexplored, highlighting a gap in the current understanding of its mechanistic actions.

These findings highlight the therapeutic potential of both POA isomers in the management of obesity and associated metabolic disorders. Beyond hepatic effects, POA has demonstrated broader metabolic benefits in animal models, including improved insulin sensitivity, reduced systemic inflammation, and attenuation of weight gain and visceral adiposity^13,14^. However, findings from human studies present a more complex picture. Elevated circulating levels POA have been positively correlated with hepatic steatosis, suggesting that POA may serve as a biomarker of hepatic *de novo* lipogenesis and metabolic dysfunction^15^. These findings raise important questions about the context-dependent effects of POA and the potential risks of dietary supplementation, particularly in individuals with pre-existing metabolic disorders. Also, most existing studies have primarily focused on the *cis* isomer or natural mixtures containing cPOA, leaving the role of tPOA less explored. Our recent research has unveiled that both isomers enhance glucose-stimulated insulin secretion (GSIS), yet they do so via distinct intracellular signaling pathways^16^. Taken together, these observations underscore the need for comprehensive, mechanistic studies to delineate the distinct roles of cPOA and tPOA in metabolic regulation. Clarifying their effects on lipid metabolism, inflammation, and liver function is essential for evaluating the therapeutic potential and safety of POA in the prevention and treatment of MASLD and other metabolic diseases.

Hepatic steatosis and insulin resistance are closely linked, and compensatory hyperinsulinemia is a hallmark of early metabolic dysfunction^17^ Glucose homeostasis is often impaired in metabolic disorders such as MASLD and type 2 diabetes. Therefore, evaluating insulin secretion alongside hepatic lipid metabolism provides a more comprehensive understanding of the metabolic impact of the tested compounds. We hypothesized that cis- and trans-palmitoleic acid isomers, as well as sea buckthorn oil could modulate lipid and cholesterol metabolism and enhance GSIS through mechanisms involving HMG-CoA reductase inhibition and Rap1a prenylation. Our research aimed to elucidate the potential implications of POA isomers and SBO in the context of MASLD and related metabolic disorders. Specifically, we investigated how *cis* and *trans* isomers of POA, along with SBO extracted from the pulp of the berries and recognized as the richest known plant source of cPOA^18^, modulate the expression of HMGCR in hepatocytes. We also examined their effect on Rap1a, a key regulatory protein involved in hepatic glucose homeostasis, cellular cholesterol. metabolism, and TAG levels. Given its unique nutritional profile, SBO may offer significant benefits in managing metabolic syndrome and MASLD. To explore these effects, we employed a model of HepG2-based hepatocellular steatosis induced by stimulation with free fatty acids (FFAs)^19^. The FFA mixture consisted of oleate to palmitate, reflecting the two most prevalent fatty acids in both the human diet and hepatic steatosis: palmitic acid (C16:0), and the monounsaturated oleic acid (OA, C18:1)^20^.

While previous studies have explored the effects of POA isomers and SBO in animal models or as components of complex dietary interventions, there remains a lack of direct comparative studies in human hepatocyte models. To our knowledge, this is the first study to systematically compare the effects of cPOA, tPOA, crude and digested SBO, and lovastatin on lipid accumulation, cholesterol metabolism, and insulin secretion in HepG2 and MIN6 cells. Furthermore, we provide novel mechanistic insights into their interaction with HMGCR and their influence on Rap1a prenylation and localization—key regulators of cholesterol biosynthesis and glucose homeostasis. This multifaceted approach enables a comprehensive evaluation of the therapeutic potential of these compounds in the context of MASLD and associated metabolic dysfunctions.

## Results

### Effects of palmitoleic acid isomers and sea Buckthorn oil on lipid accumulation and viability in HepG2 cells

**Fig. 1:**
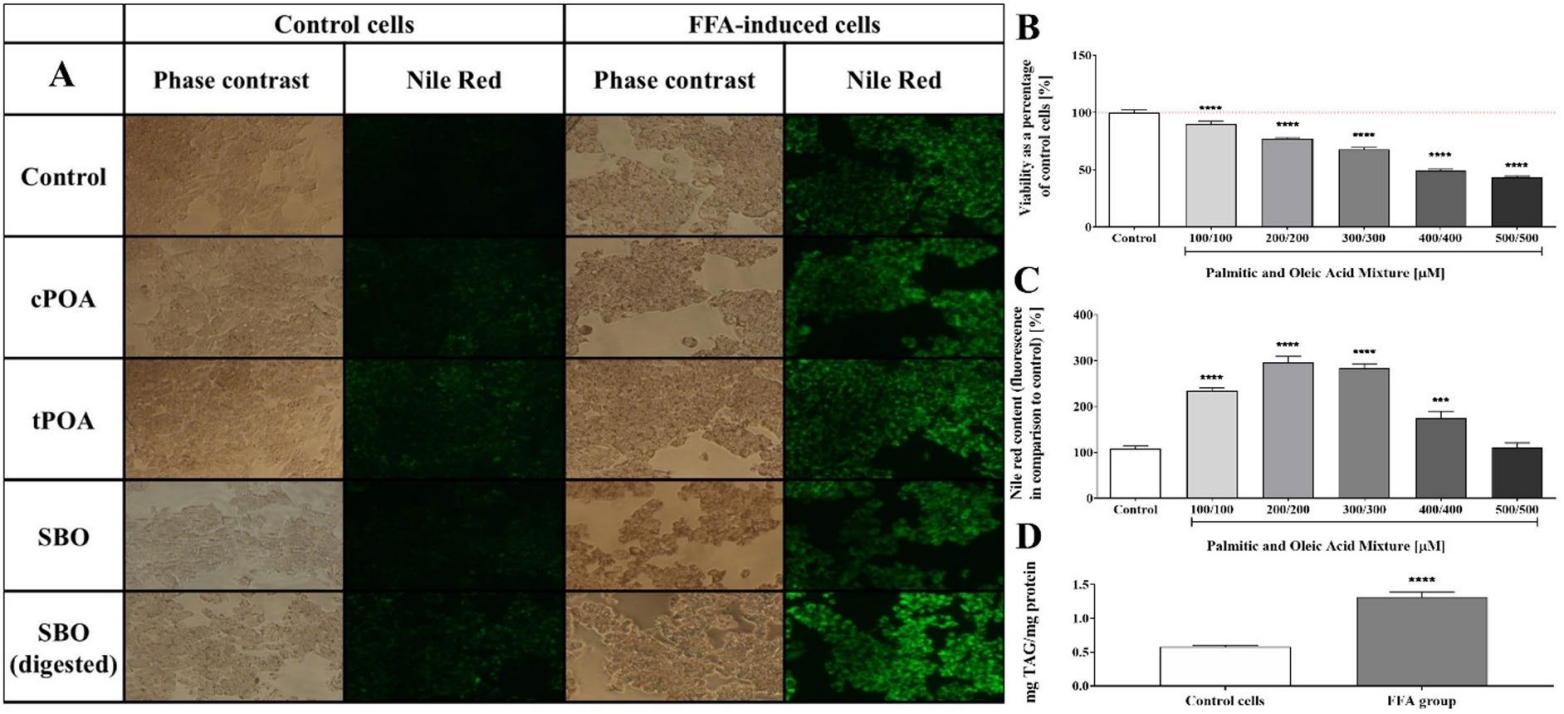
Impact of a mixture of palmitic acid (PA) and oleic acid (OA) used at various concentrations on lipid accumulation and viability in HepG2 cells. A: Representative images of HepG2 cells stained with Nile Red, captured under both visible and fluorescent light (Ex: 482/35, Em: 536/40). B: Cellular viability assessed after 24-h incubation with PA/OA mixtures. C: Quantitative analysis of intracellular lipid content using Nile Red staining. D: TAG accumulation in control and steatotic cells (300 µM PA/OA) measured with Triglyceride Colorimetric Assay Kit. The results are representative of at least five independent biological replicates (*n* = 5). The bars represent the means ± SEM. ***p < 0.001, ****p < 0.0001 versus control. Images of HepG2 cells under visible and fluorescent light (Ex: 482/35, Em: 536/40). cPOA, *cis*-palmitoleic acid; FFA, free fatty acid; SBO, sea buckthorn oil; TAG, triacylglycerol; tPOA, *trans*-palmitoleic acid.

In this study, we utilized the HepG2 cell line, a widely accepted model for studying liver metabolism, to induce an in vitro model of hepatic steatosis. Based on established methodologies^21,22^, we treated cells with a mixture of palmitic acid and oleic acid at various concentrations (100, 200, 300, 400, and 500 µM in a 1:1 molar ratio) for 24 h. Compared to the control group, which exhibited a steatosis-free phenotype, the FFA-treated cells showed a marked accumulation of lipid droplets (Fig. 1A). Quantitive analysis using Nile Red staining confirmed a significant increase in neutral lipids, which include but are not limited to triacylglycerols (Fig. 1B). Notably, lipid accumulation increased sharply at 200 and 300 µM of the OA/PA mixture, reaching approximately threefold the levels observed in control cells. However, at higher concentrations, a rapid decline in the Nile Red signal was observed, likely due to the hepatotoxic effects of excessive fatty acid exposure (Fig. 1C). This observation aligns with previous findings that hepatocytes isolated from fatty livers demonstrate reduced cell viability, likely due to cellular stress, disrupted metabolic function, increased susceptibility to damage and subsequent apoptosis^23^. Consistent with these observations, an enzymatic assay revealed that the TAG level in the steatosis group was significantly elevated compared to the control group, with values of 1.32 mg TAG/mg protein versus 0.58 mg TAG/mg protein, respective (Fig. 1D). Based on this preliminary dose–response experiments and available literature, a mixture of 300 µM oleic acid (OA) and 300 µM palmitic acid (PA) was selected to induce steatosis. The selected concentration ensured a balance between effective lipid accumulation and acceptable cell survival. Importantly, previous studies have shown that combined OA and PA treatment better mimics the pathophysiological state of hepatic steatosis in vitro compared to the use of either fatty acid alone^20^.

After establishing the steatosis model, we proceeded to assess the potential cytotoxicity of the tested compounds by assessing HepG2 cell viability following exposure to varying concentrations (5-100 µM). The compounds under investigation included cPOA, tPOA, SBO, and digested SBO. Since dietary oils mainly consist of triacylglycerols, which are hydrolyzed to free fatty acids and monoacylglycerols during digestion before absorption into the bloodstream, SBO was subjected to in vitro digestion with pancreatic lipase before testing. This step aimed to simulate better the physiological conditions under which SBO-derived lipids exert their biological effects.

**Fig. 2:**
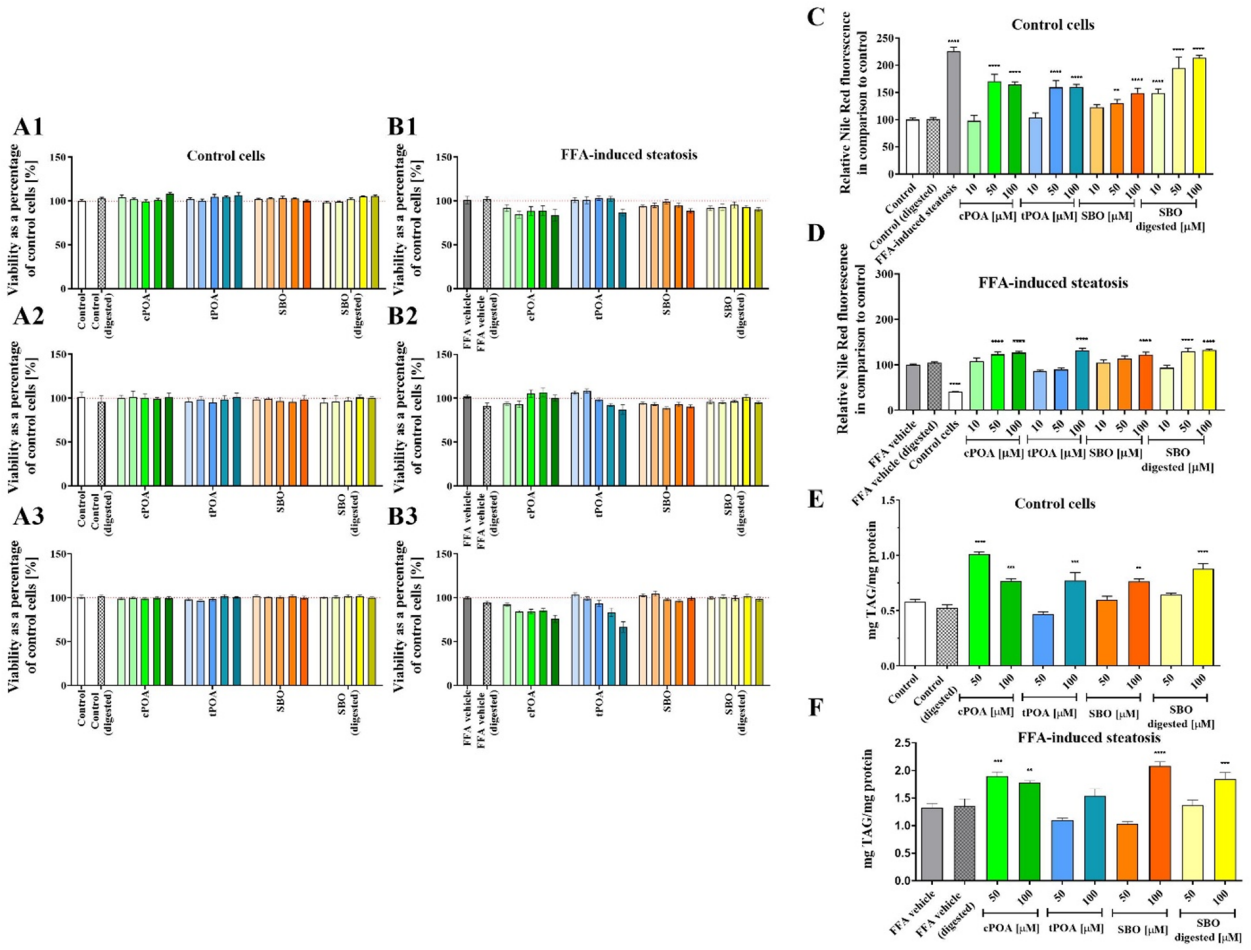
Effects of cPOA, tPOA, SBO, and digested SBO on viability and lipid accumulation in control and FFA-induced steatostic HepG2 cells. A: Viability of control cells treated with increasing concentrations (5–100 µM) of the tested compounds. For SBO, concentrations were adjusted based on POA content. B: Viability of cells with FFA-induced steatosis (300 µM PA/OA) following treatment with the same concentration range of compounds. Viability was assessed at 24 (A1, B1), 48 (A2, B2), and 72 (A3, B3) hours; bar shading intensity corresponds to increasing concentrations: 5, 10, 25, 50, and 100 µM. C: Quantitative detection of lipid accumulation in control cells using Nile Red fluorescence staining. D: Quantitative detection of lipids with Nile Red staining in steatosis-induced cells (300 µM PA/OA). E: TAG accumulation in control cells measured with a Triglyceride Colorimetric Assay Kit. F: TAG accumulation in steatotic cells measured with a Triglyceride Colorimetric Assay Kit. Nile Red staining and TAG detection were conducted after 24 h of incubation with the tested compounds. Data represent at least 8 independent biological replicates (*n* = 8). The bars represent the means ± SEM. **p < 0.01, ***p < 0.001, ****p < 0.0001 versus control. cPOA, *cis*-palmitoleic acid; FFA, free fatty acid; OA/PA, oleic acid/palmitic acid; SBO, sea buckthorn oil; TAG, triacylglycerol; tPOA, *trans*-palmitoleic acid.

The concentration of palmitoleic acid in the SBO sample was estimated at 30.35 ± 0.51 g/100 g oil^24^. Our results indicated that none of these compounds exhibited cytotoxic effects on the cells across the studied concentration range following incubation of 24, 48, and 72 h (Figs. 2A1-2B3). Furthermore, treatment of FFA-induced steatotic cells ( 300/300 µM PA/OA) with these compounds for 24 h did not result in reduced metabolic activity (Fig. 2B1). Upon extending the incubation period to 72 h, we observed a slight, concentration-dependent decrease in cell viability for cPOA and tPOA. Specifically, at the highest concentration tested (100 µM), cell viability decreased to 75.9% for cPOA and 66.6% for tPOA, (Fig. 2B3).

Having confirmed that the tested compounds did not exert a cytotoxic effects on HepG2 cells under 24-hour exposure, we examined their impact on intracellular lipid accumulation using Nile Red fluorescence staining complemented by an enzymatic assay. Our observations revealed a concentration-dependent increase in intracellular lipid accumulation for nearly all tested compounds, irrespective of the cell culture conditions or analytical method applied (Figs. 2C-F). However, this effect was significantly diminished in steatosis-induced cells, likely due to their limited capacity to incorporate additional fatty acids. In the Nile Red assay, treatment with cPOA, tPOA, SBO, and digested SBO at 50 and 100 µM significantly increased the fluorescence signal in non-steatotic cells. Only digested SBO affected cells, even at a 10 µM concentration. The significantly elevated level of signal varied from 130% for SBO (50 µM) to 213% for digested SBO (100 µM) (Fig. 2C). In contrast, steatosis-induced cells exhibited a more modest increase in lipid accumulation, not exceeding 133%. Interestingly, tPOA slightly reduced the Nile Red fluorescence signal at 10 and 50 µM, indicating a potential lipid-lowering effect under these conditions.

In control cells HepG2 cells, treatment with 100 µM concentration of cPOA, tPOA, SBO, and digested SBO resulted in a moderate increase in the TAG content, reaching 0.77, 0.77, 0.76, and 0.88 mg/mg of protein, respectively (Fig. 2E). In steatosis-induced cells, the response to the added compounds was more variable. The highest TAG content was observed for 100 µM of SBO (2.08 mg/mg protein), followed by 50 µM cPOA (1.90 mg/mg protein), 100 µM digested SBO (1.85 mg/mg protein), and 100 µM cPOA (1.78 mg/mg protein), all exceeding the control value (1.32 mg/mg protein). Notably, tPOA at the highest concentration did not significantly elevate TAG content, and 50 µM of tPOA and SBO slightly reduced TAG concentration (1.10 and 1.03 mg/mg protein, respectively) (Fig. 2F). These findings suggest that while most compounds promote lipid accumulation in steatotic cells, tPOA may exert a modest lipid-lowering effect at lower concentrations.

### Effects of palmitoleic acid isomers and sea Buckthorn oil on the cholesterol level in HepG2 cells

**Fig. 3:**
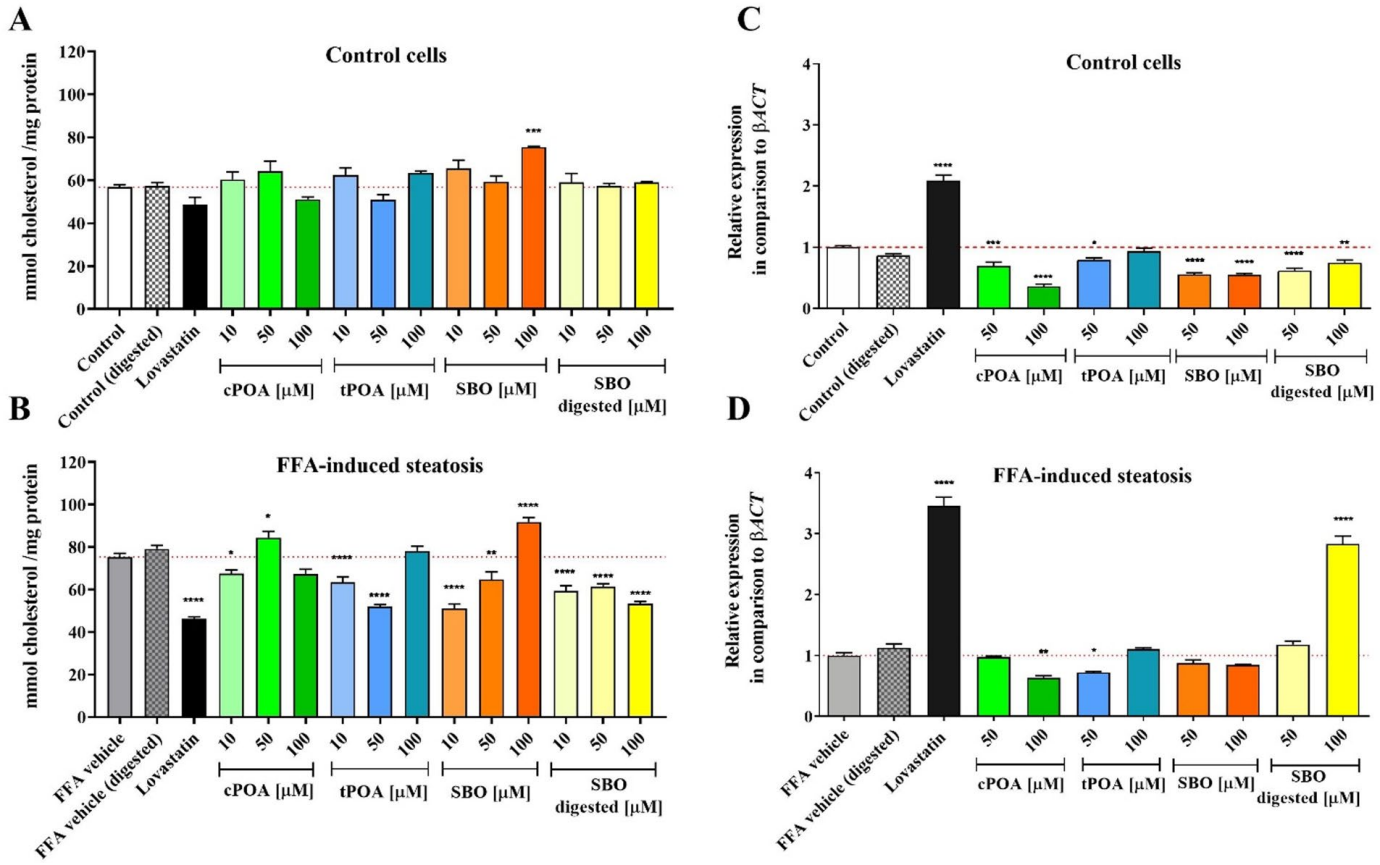
Effects of cPOA, tPOA, SBO, and digested SBO on intracellular cholesterol content (A, B) and *HMGCR* gene expression (C, D) in control (A, C) and FFA-induced steatotic (B, D) HepG2 cells. Cells were exposed to indicated concentrations of compounds or 5 µM lovastatin. Results represent at least 3 independent biological samples with 3 repeats each (*n* = 3). The bars represent the means ± SEM. *p < 0.05, **p < 0.01, ***p < 0.001, ****p < 0.0001 versus control. β*ACT*, β-aktyna; cPOA, *cis*-palmitoleic acid; FFA, free fatty acid; OA/PA, oleic acid/palmitic acid; SBO, sea buckthorn oil; tPOA, *trans*-palmitoleic acid.

In the subsequent phase of our study, we investigated the impact of POA isomers and SBO supplementation on the intracellular cholesterol levels in HepG2 cells. In non-steatotic (control) cells, treatment with cPOA, tPOA, SBO, and digested SBO at concentrations ranging from 10 to 100 µM generally did not result in significant changes in cholesterol levels. An exception was observed with 100 µM of SBO, which elevated the cholesterol concentration to 75.4 mmol/mg of protein, compared to the control value of 56.8 mmol/mg of protein (Fig. 3A). The results were particularly noteworthy in steatosis-induced cells. As a reference, 5 µM lovastatin, a known HMG-CoA reductase inhibitor, decreased cholesterol quantity to 46.2 mmol/mg of protein, compared to 75.3 mmol/mg of protein in untreated steatotic control cells. Notably, lovastatin had no significant effect on normal cells‘ cholesterol levels. Among the tested compounds, cPOA exhibited a biphasic effect: 10 µM decreased cholesterol quantity to 67.5 mmol/mg, whereas 50 µM increased it to 84.4 mmol/mg. In contrast, tPOA significantly reduced cholesterol at 10 µM (63.4 mmol/mg) and 50 µM (52.0 mmol/mg protein), but not at 100 µM. SBO showed a similar trend, with lower concentrations decreasing cholesterol production, while 100 µM led to a marked increase (91.8 mmol/mg protein). In contrast, digested SBO consistently reduced cholesterol levels across all analyzed concentrations (Fig. 3B).

We further examined the potential impact of tested compounds on the expression of HMGCR using RT-qPCR. As expected, lovastatin significantly upregulated HMGCR expression, increasing it by approximately 2- and 3.5-fold in normal and steatosis-induced cells, respectively. In normal HepG2 cells, HMGCR expression was reduced by almost all tested compounds, with the exception of 100 µM tPOA (Fig. 3C). In steatosis-induced cells, only 100 µM cPOA (0.6-fold3) and 50 µM tPOA (0.72-fold) downregulated HMGCR expression. Remarkably, a 100 µM concentration of digested SBO led to a strong upregulation of HMGCR expression (2.8-fold) (Fig. 3D).

### Molecular docking: interactions between palmitoleic acid isomers and HMG-CoA reductase

**Fig. 4:**
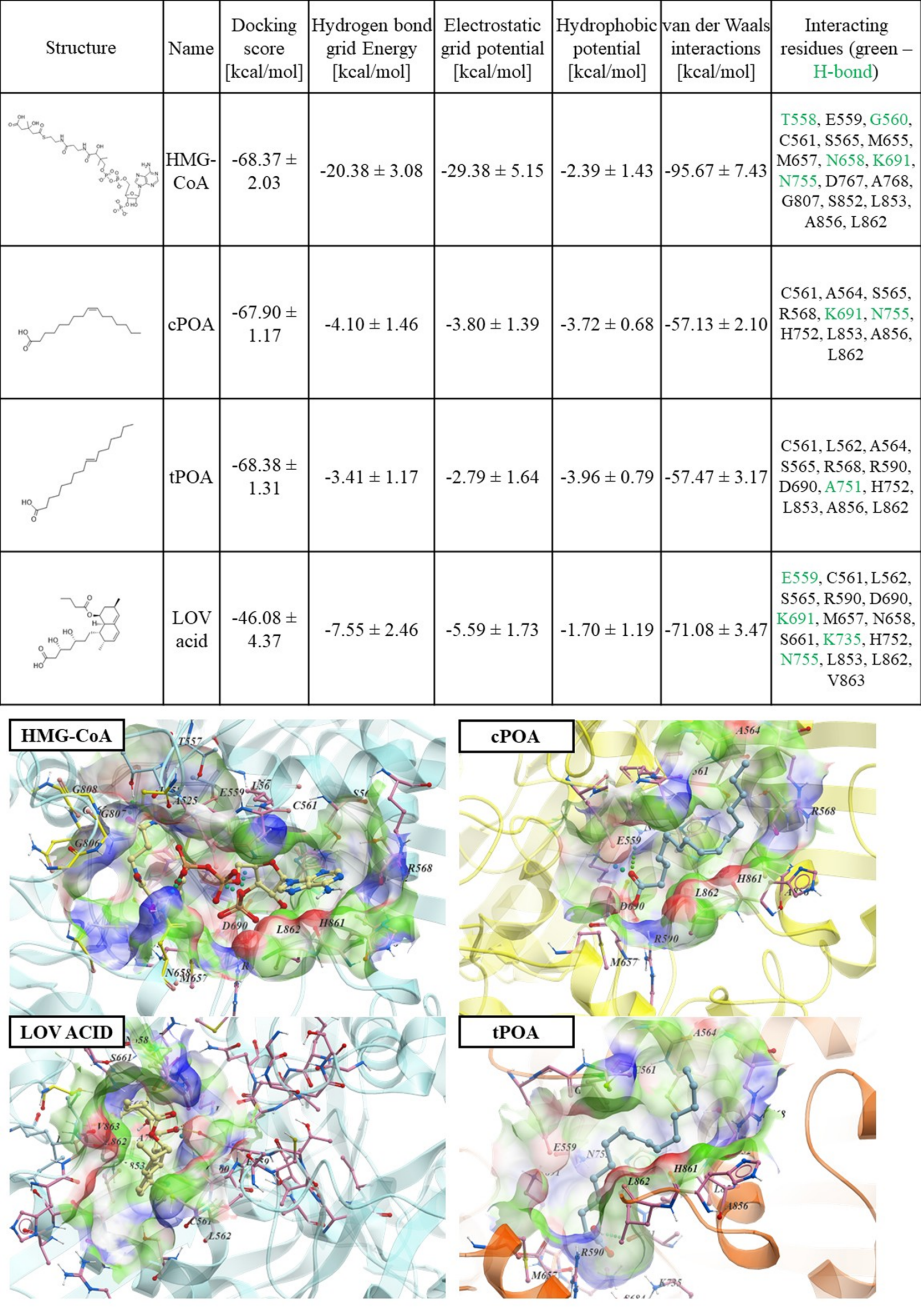
Molecular docking of cPOA, tPOA, HMGCoA, and lovastatin acid into HMGCoA reductase. Mean score and energy value were presented with the means ± SD from at least 10 experiments. The location of the molecule in the catalytic pocket, with the most important amino acid residues within the binding pocket, is shown on the bottom. The neighbouring amino acid residues are listed in the table, with those forming hydrogen bonds with the molecule highlighted in green. cPOA, *cis*-palmitoleic acid; HMG-CoA, 3-hydroxy-3-methylglutaryl coenzyme A; LOV ACID, lovastatin acid; tPOA, *trans*-palmitoleic acid.

A promising strategy to decrease cholesterol content without altering gene expression involves the direct inhibition of HMG-CoA reductase^25^ To xplore this possibility, we performed molecular docking simulations to assess the binding potential of POA isomers to the enzyme’s catalytic site. For comparison, HMG-CoA (the natural substrate) and lovastatin acid (the active form of the statin drug) were also under identical conditions. Although the calculated individual energies such as hydrogen bonding, electrostatic, hydrophobic, and van der Waals potentials, did not differ significantly between ligands, the overall energy state (final score) for both POA isomers yielded a docking score similar to that of HMG-CoA (Fig. 4). This suggests that both cPOA and tPOA are capable of binding within the catalytic pocket. Interestingly, the lovastatin acid-reductase complex yielded an even lower score, consistent with its potent inhibitory activity^26^.

Istvan et al. (2001) reported that various statins, including compactin, simvastatin, fluvastatin, cerivastatin, atorvastatin, and rosuvastatin, bind to HMGCR^27^. In all cases, statins efficiently blocked the substrate from entering the catalytic pocket by occupying the HMG moiety site. These statins form polar interactions within HMG-binding sites with residues such as S684, D690, K691, and K692, and nonpolar interactions with L562, V683, L853, A856, and L857. Notably, minor differences existed in the binding of the analyzed statins. For instance, rosuvastatin created additional polar interactions with R568 and S565; atorvastatin also interacted with S565. Lovastatin acid in particular, binds deeply within the catalytic pocket, forming hydrogen bonds with E559, K691, K735 and N755 while its nonpolar fragments were surrounded by residues such as C561, L562, S565, R590, D690, M657, N658, S661, H752, L853, L862 and V863 (Fig. 4). The acid form of lovastatin resembles the biding pattern of other statins documented by the crystallography method and proves its ability to inhibit HMGCR^25^.

Docking simulations revealed that both POA isomers occupy the HMG-CoA reductase’s catalytic center, albeit in slightly different positions. The *cis* isomer formed two hydrogen bonds with K691 and N755 anchoring it at the HMG-binding site, while the *trans* isomer interacted with A751 suggesting an alternative binding mode. The nonpolar tail of both fatty acids extends through the catalytic pocket, generating hydrophobic and van der Waals interactions with residues such as C561, A564, S565, R568, H752, L853, A856 and L862 (Fig. 4, Supplementary Fig. S1). These findings strongly the hypothesis that both analyzed acids can bind and probably block the catalytic pocket of HMGCR.

HMG-CoA, the natural substrate of HMGCR, establishes polar interactions with T558, G560, N658, K691 and N755. The CoA part of the molecule is also stabilized through interactions with C561 and L853, R565 and K722, and Y476^28^. These interactions underscore the critical role of polar residues (R590, D690, K691, K692) in substrate binding and enzymatic activity (Supplementary Fig. S1). The docking-derived model largely confirmed the significance of amino acid residues previously identified in crystallographic studies as critical for HMG-CoA binding. This consistency supports the reliability of the docking approach and highlights the conserved nature of the binding interactions within the active site. The ability of palmitoleic acid isomers to engage with several of these identical residues suggests that they may competitively bind to the catalytic pocket and modulate HMGCR activity, thereby influencing cholesterol biosynthesis.

### The influence of palmitoleic acid isomers and sea Buckthorn oil on Rap1 prenylation and cellular localization

Statin therapy has been associated with an increased risk of newly diagnosed Type 2 Diabetes, partly due to decreasing geranylgeranyl pyrophosphate (GGPP) levels and subsequently inhibiting Rap1a GTPase activity. Statins impair Rap1a prenylation and its membrane localization in hepatocytes by inhibiting HMGCR^28^. Given this mechanism, we investigated whether POA isomers and SBO exert similar effects on Rap1a prenylation, the protein modified by geranylgeranyl transferase type I (GGT-I). Lovastatin was employed as a positive control.

To assess Rap1a localization, we performed the fractionation of cellular lysates followed by the immunochemical detection by Western blot analysis. The Mem-PER™ Plus Membrane Protein Extraction Kit was used to isolate two distinct fractions: a cytosolic fraction, containing non-prenylated Rap1a, and a membrane fraction, containing prenylated Rap1a anchored via farnesyl or geranylgeranyl residues. This method, previously validated in our labolatory^29^ avoids the limitations of hydrophobicity-based phase separation and ensures reliable detection of prenylated proteins.

**Fig. 5:**
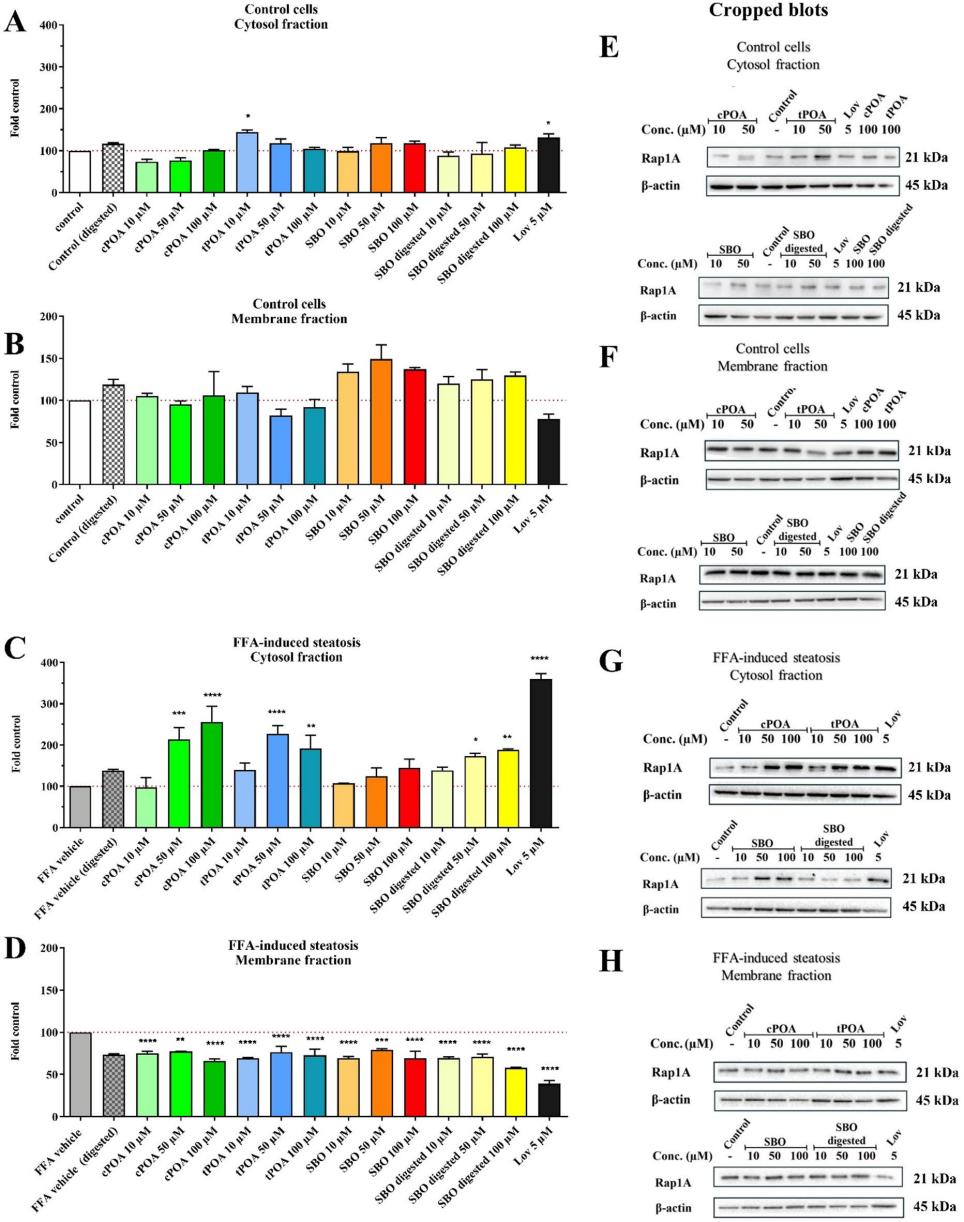
Effect of cPOA, tPOA, SBO, and SBO digested on Rap1A prenylation in HepG2 cells. Control (A, B and E, F) and FFA-induced steatotic cells (C, D and G, H) were exposed to indicated concentrations of compounds or 5 µM lovastatin (Lov) for 48 h. Subsequently, cells were lysed and isolation of cytosolic and membrane proteins was performed. Cytosolic fractions containing unprenylated proteins were western blotted for Rap1A and β-actin (A, C and E, G), and the immunoblot bands were quantified by densitometry analysis, normalized to β-actin, and presented as a percentage of controls (B, D and F, H). Lovastatin was used as a positive control. Treatment of cells with lovastatin should prevent the Rap1A accumulation at the membrane. Data represented mean ± SEM from at least three independent experiments (*n* = 3); *p ≤ 0.05, **p ≤ 0.01, ***p ≤ 0.001, and ****p ≤ 0.0001. Cropped gels are presented here; full-length gels and blots without cropping are included in the Supplementary Information file. cPOA, *cis*-palmitoleic acid; FFA, free fatty acid; OA/PA, oleic acid/palmitic acid; SBO, sea buckthorn oil; tPOA, *trans*-palmitoleic acid.

We found that in non-steatotic HepG2 cells, treatment with cPOA, tPOA, SBO, and digested SBO did not significantly Rap1a distribution between cytosolic and membrane fractions. However, lovastatin lowered membrane Rap1a level by 23% while increasing its cytosolic accumulation (Fig. 5A, E, B and F). Interestingly, the effects were more pronounced in FFA-induced steatotic cells. Lovastatin’s impact was more substantial, leading to a 3.5-fold increase in cytosolic Rap1 and a 60% reduction in membrane-bound Rap1. POA isomers also showed stronger activity under steatotic conditions, particularly at concentrations of 50 and 100 µM, where they increased cytosolic Rap1 levels. A similar, though slightly weaker, effect was observed for SBO. In contrast, membrane-bound Rap1 decreased following treatment with cPOA, tPOA, and SBO (Fig. 5C, G, D and H). These findings suggest that under steatotic conditions, POA isomers and SBO may partially mimic statin-like effects on Rap1a prenylation and localization, potentially contributing to their metabolic regulatory properties.

### The influence of palmitoleic acid isomers and sea Buckthorn oil on glucose-stimulated insulin secretion

**Fig. 6:**
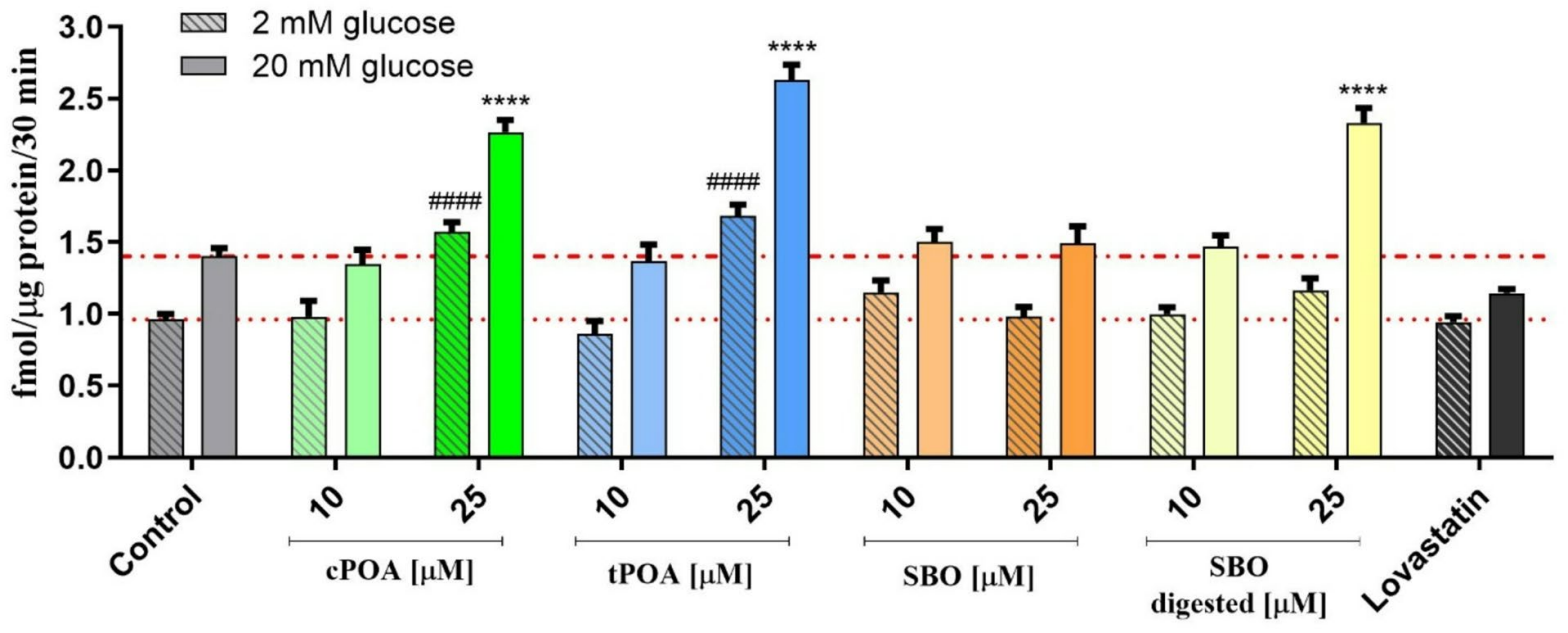
Effect of cPOA, tPOA, SBO, and SBO digested on glucose-stimulated insulin secretion (GSIS) in MIN6 cells. Cells were exposed to indicated concentrations of compounds or 5 µM lovastatin for 24 h. Data represented mean ± SEM from at least three independent experiments (*n* = 3); ^####^*p* ≤ 0.0001 versus 2 mM glucose control, ****p ≤ 0.0001 versus 20 mM glucose control. cPOA, *cis*-palmitoleic acid; SBO, sea buckthorn oil; tPOA, *trans*-palmitoleic acid.

To evaluate the effects of POA isomers and SBO on glucose-stimulated insulin secretion, MIN6 pancreatic β-cells were treated with POA isomers, both crude and digested SBO, and lovastatin under low (2 mM) and high (20 mM) glucose conditions. Insulin levels were quantified in the conditioned media and normalized to total protein content. In control cells, insulin secretion increased from 0.960 fmol/µg under low glucose to 1.402 fmol/µg under high glucose conditions, confirming glucose responsiveness (Fig. 6). Our findings reveal that both tPOA and cPOA enhanced GSIS, with tPOA exhibiting a slightly stronger stimulatory effect. Interestingly, digested SBO significantly stimulated insulin secretion, a phenomenon not observed with its undigested variant. The stimulatory activities on insulin secretion were detected more pronounced under high glucose, consistent with a glucose-dependent mechanism, especially in the case of SBO digested. Conversely, the introduction of lovastatin appeared to reduce insulin release, but only at a high glucose concentration.

## Discussion

Nutrition interventions have emerged as a cornerstone in the management of MASLD. Numerous observational studies have shown a link between this disorder and diets high in saturated fatty acids (SFAs) and low in polyunsaturated fatty acids (PUFAs), regardless of the total caloric intake. Also, a diet rich in monounsaturated fatty acids has been shown to significantly reduce the hepatic fat content^30^. Among the most notable dietary components, n-3 PUFAs have demonstrated the ability to lower plasma triacylglycerols and lipoprotein concentrations in individuals with hypertriglyceridemia and healthy subjects. The mechanism behind this phenomenon can be traced back to the inhibitory action of PUFAs on HMG-CoA reductase. PUFAs, with their potent inhibitory action, can effectively suppress the activity of this enzyme, thereby controlling the rate of cholesterol synthesis^31^. 

^13,32,33,14,15^Our objective was to explore the impact of POA isomers and sea buckthorn oil, the most abundant plant-based source of POA^18^, on the TAG and lipid content in hepatocytes. For this purpose, we employed an FFA-induced steatosis model using HepG2 cells. Our findings suggest that both cPOA and tPOA, as well as SBO and digested SBO, are generally non-toxic to HepG2 cells. These compounds did not reduce the metabolic activity of the cells even under steatosis-induced conditions. Only high concentrations of cPOA and tPOA reduced cell viability after prolonged exposure. Importantly, this supports the potential therapeutic application of these compounds without causing significant hepatocellular damage. Nezhad et al. showed that while a concentration greater than 1 mM for cPOA reduced the viability of hepatocytes, tPOA revealed an opposite effect and increased cell survival^34^.

Regrettably, there is a scarcity of comprehensive reports in the literature that elucidate the effect of POA isomers on lipid metabolism in human cells. Most existing research has focused on rodent models in which POA supplementation is administered. For instance, studies with db/db mice, a model characterized by disrupted lipid metabolism, obesity, hypertension, and diabetes indicated that POA significantly reduced serum total cholesterol and high-density lipoprotein cholesterol concentrations and decreased the fat index. Conversely, the cPOA group exhibited an upward trend in the fat index. cPOA also reduced serum total cholesterol concentrations, high-density lipoprotein cholesterol (LDL-C), and liver-free cholesterol, although the effect was not statistically significant^12^. In another study, the consumption of tPOA and HFD by mice C57BL/6 led to a reduction in liver TAG content^14^. However, the authors did not include the *cis* isomer in this study, leaving a gap in comparative analysis. Similarly, Nezhad et al. confirmed that tPOA treatment significantly reduced lipid accumulation in HepG2 cells compared to PA, and co-treatment with tPOA attenuated PA-induced steatosis, especially at lower doses^34^. These findings underscore the potential therapeutic role of tPOA in lipid-related disorders, yet the absence of cPOA in these studies limits our understanding of its regulatory effects.

This gap was partially addressed by Huang et al., who compared the effects of cPOA and tPOA on cholesterol metabolism in mice with HFD-induced hypercholesterolemia. Both isomers were found to reduce hepatic and serum cholesterol levels, albeit through different mechanisms. The study identified HMGCR and LXR as targets influenced by both isomers. However, the specific pathways mediating these antihypercholesterolemic effects were not fully elucidated^12^. Moreover, since the publication of Huang et al.‘s study, no further investigations have explored the effects of cPOA and tPOA in human cellular models.

To address this knowledge gap, our study employed both normal and steatotic human HepG2 cells. We observed that treatment with 100 µM concentrations of cPOA, SBO, and digested SBO led to accumulation of TAGs in both cell types, with, with a more pronounced effect in normal cells. Notably, the two POA isomers exhibited distinct effects: cPOA consistently increased lipid accumulation across all tested concentrations concentrations, whereas tPOA, particularly at lower concentrations, marginally reduced TAG levels.

Another critical aspect of lipid metabolism is the level of cholesterol. While both tPOA and cPOA have previously been shown to reduce cholesterol levels in mice fed a HDF^12^, their effects on human cells remain largely unexplored. In our study, we observed that in normal HepG2 cells, even high concentrations of the tested compounds had minimal impact on cholesterol levels, except for 100 µM SBO. However, in steatotic cells, tPOA, SBO (at concentrations of 10 and 50 µM), and digested SBO (10–100 µM) significantly reduced the amount of cholesterol. In contrast, cPOA only demonstrated a cholesterol-lowering effect at 10 µM.

Cholesterol is produced via the mevalonate pathway, beginning with acetyl-coenzyme A (Ac-CoA) and involving nearly 30 enzymatic reactions. The rate-limiting step is the reduction of HMG-CoA to mevalonate, catalyzed by HMGCR, making the regulation of HMGCR expression and activity a critical control point in cholesterol biosynthesis. An increased cellular cholesterol level inhibits HMGCR expression^35^.

^36^Our studies confirmed that lovastatin, a known HMGCR inhibitor, significantly reduced cholesterol production in steatotic cells. Blocking the HMG-CoA reductase function triggered a compensatory feedback loop, increasing HMGCR expression to compensate for the required deficiencies. This phenomenon has been previously documented. For example, atorvastatin treatment upregulated HMGCR mRNA levels in DU-145 cells in a dose-dependent manner^36^, and similar effects were observed with simvastatin in HepG2 cells^37^. In our experiments, the expression of HMGCR in normal cells decreased with almost all tested compounds, except for 100 µM tPOA. In steatotic cells, only 100 µM cPOA and 50 µM tPOA lowered the HMGCR level. Although the influence of fatty acids on HMGCR expression and activity has been studied, the mechanisms remain incompletely understood. Fatty acids can modulate HMGCR activity depending on the degree of saturation and chain length. For instance, in C6 glioma cells, stearic (C18:0) and arachidonic (C20:4, ω-6) acids have been shown to decrease HMGCR activity similarly, while *cis*-oleic acid (C18:1) exhibited the most potent inhibitory effect^38^. In HepG2 cells, PUFAs such as eicosapentaenoic (C20:5, ω-3, EPA) and arachidonic acids reduced HMGCR expression^39^. Interestingly, co-treatment with EPA and lovastatin enhanced the regulatory effect in the same cells, although the underlying molecular mechanisms remain unclear^31^. DeBose-Boyd et al. proposed that unsaturated fatty acids (UFAs) inhibit the proteolytic activation of sterol regulatory element-binding proteins (SREBPs). Unlike saturated fatty acids, UFAs bind to the UAS domain of Ubxd8, inducing its polymerization. This process of polymerization blocks interaction with Insig-1 and prevents its degradation. This polymerized form prevents interaction with Insig-1, stabilizing it and allowing it to bind the Scap-SREBP complex, thereby blocking SREBP activation^40^. This mechanism may explain the observed reduction in HMGCR expression in our study, though further studies are needed to confirm this.

Interestingly, similar to lovastatin, 100 µM digested SBO also induced HMGCR upregulation, accompanied by a concentration-dependent decrease in cholesterol. This phenomenon may reflect a feedback inhibitory loop in which reduced cholesterol levels activate SREBP-2, which in turn upregulates HMGCR to restore homeostasis. This regulatory loop has been observed in HepG2 cells treated with palmitic acid, where both HMGCR expression and activity increased. SREBP-2 is transcriptionally regulated by specific protein 1 (Sp1), and silencing Sp1 via siRNA effectively suppressed SREBP-2 and HMGCR expression in PA-treated HepG2 cells^41^ .

It is worth to underline that HMGCR is a rate-limiting enzyme not only in cholesterol biosynthesis but also in the production of isoprenoids. Isopentenyl pyrophosphate (IPP), a product of the mevalonate pathway, is used to synthesize farnesyl pyrophosphate (FPP), which diverges into either squalene synthesis for cholesterol production or GGPP for prenylation. Many membrane-associated regulatory proteins require lipid modifications for proper localization and function. Among these, protein prenylation, characterized by the attachment of a farnesyl or geranylgeranyl anchor to conserved cysteine residues at the C-terminus, stands out among post-translational modifications. Geranylgeranyltransferase type I (GGTase-I) catalyzes the transfer of a single isoprenoid chain from GGPP to substrate proteins such as Rho, Ral, and Rap family of small GTPases, anchoring them to cellular membranes and enabling their biological activity^42^.

The contribution of Rap1a in metabolic regulation has been increasingly recognized, particularly in the context of lipid and glucose homeostasis. Activating Rap1a has been shown to reduce plasma LDL levels by downregulating proprotein convertase subtilisin-kexin type 9 (PCSK9)^43^. Statins, widely used to lower plasma LDL, achieve this by increasing LDL receptor expression^44^. Besides, in AML-3 cells, lovastatin impaired Rap1a prenylation, and ERK1/2 phosphorylation was completely abolished, triggering apoptosis. These effects were reversible only with GGPP supplementation, underscoring the central role of isoprenoid biosynthesis in protein prenylation. Similar dose-dependent effects were observed in multiple myeloma cell lines, where lovastatin inhibited prenylation of Rap1a and DnaJ protein^45^. However, statins also promote hepatic gluconeogenesis and elevate glucose production through mechanisms independent of LDLR and PCSK9. One key pathway involves the inhibition of HMG-CoA reductase, which reduces the synthesis of GGPP, a critical for activating Rap1a^46^. In hepatocytes and human liver tissue, statin treatment leads to reduced levels of active-Rap1a. This inhibition mimics the effects of direct Rap1a suppression, resulting in enhanced hepatic gluconeogenesis and raised fasting blood glucose in obese mice. Notably, supplementation with geranylgeraniol, a precursor for geranylgeranyl isoprenoids, restores Rap1a activity and mitigates the glucose intolerance induced by statins. Mechanistically, Rap1a activation promotes actin polymerization, which in turn suppresses gluconeogenesis via Akt-mediated FoxO1 inhibition. Therefore, Rap1a plays a crucial role in maintaining hepatic glucose homeostasis, and its inhibition, through the reduction of geranylgeranyl isoprenoids, contributes to the glucose intolerance caused by statins^28^.

Our study is the first to demonstrate that POA isomers and SBO can modulate Rap1a protein levels and its subcellular distribution in HepG2 cells. Molecular modelling revealed that both POA isomers occupy the catalytic site of HMG-CoA reductase, suggesting a direct interaction with this key enzyme in the cholesterol synthesis pathway. In HepG2 cells not treated with an OA/PA mixture, these compounds did not significantly alter cytosolic or membrane-bound Rap1a levels. However, in steatosis-induced cells, the effects were more pronounced. Both POA isomer, particularly at 50 and 100 µM, markedly increased cytosolic Rap1 levels, while reducing its membrane-associated form. SBO exhibited a similar effect, albeit slightly weaker. These findings suggest that POA isomers and SBO may influence the localization of Rap1,

and, consequently, its functional activity in metabolic pathways.

Our findings suggest that POA isomers and SBO, like statins, influence key metabolic processes in the liver. However, unlike statins, which impair glucose tolerance by inhibiting Rap1a and GSIS,^29^cPOA, tPOA, or SBO appear to exert beneficial effects. We have demonstrated that these compounds enhance GSIS, potentially compensating the hyperglycemic effect associated with Rap1a inhibition. Our previous study showed that SBO stimulates insulin secretion via FFAs released during digestion, which act as as ligands of pancreatic G protein-coupled receptors (GPCRs)^24^. This effect could have important implications for treating conditions characterized by glucose intolerance. Elevated cholesterol levels can impair insulin secretion^47^ while palmitoleic acid lowers cholesterol both in mice^12^ and steatotic hepatic cells, leading to enhanced GSIS. One proposed mechanism involves cholesterol-mediated hydrolysis of phosphatidylinositol 4,5-bisphosphate (PIP2), which disrupts actin organization and membrane depolarization—key steps in insulin secretion^48^. On the other hand, lovastatin significantly inhibited GSIS in our study, highlighting a potential drawback of statin therapy in glucose-intolerant individuals. The comparative design of our study is a key strength, as we evaluated both cis- and trans-palmitoleic acid, SBO, and its digested form in a human hepatocyte model of steatosis and extended our analysis to pancreatic β-cells. Importantly, we demonstrated for the first time that these compounds modulate Rap1a prenylation and localization and interact with HMG-CoA reductase in a manner similar to statins.

It is worth mentioning that both the therapeutic and toxic effects attributed to cPOA found in SBO can be modulated by the presence of other bioactive lipids, acting synergistically or independently. These compounds include carotenoids (mainly precursors of vitamin A), tocopherols (with dominant α-tocopherol) and sterols (predominantly β-sitosterol and 24-methylenecycloartanol). However, these constitute a very small portion of the lipids found in SBO: approximately 1.18% sterols, 0.12% carotenoids, and 0.09% tocopherols, respectively^18^. An additional problem related to unsaponifiable compounds is the significant variation in content depending on the cultivation location, genotype, and extraction method^49^. Our previous studies compared five sea buckthorn cultivars grown in Europe in terms of lipid component content. The tested cultivars differed significantly in their levels of carotenoids, tocols, and sterols, while the fatty acid composition was relatively constant^18^. Additionally, we characterized five SBO variants prepared using different extraction methods. They differed slightly in fatty acid content, with overall values ranging from 87.81 to 88.97 g per 100 g of sample but significantly differed in unsaponifiable compound content^24^. FFA, accounting for as much as 88% of the total lipid content, thus became the focus of our research. As mentioned earlier, SBO was subjected to in vitro lipolysis, mimicking the natural digestive process. We previously demonstrated the validity of this process in studies on pancreatic β cells. Digested sea buckthorn oleosomes reduced the viability of the mouse MIN6 cell line and the human EndoC-βH1 cell line, and the toxicity observed with digested sea buckthorn preparations resulted from the released FFAs^49^. In studies on GSIS, undigested SBO did not affect insulin secretion. To confirm the role of fatty acids in stimulating insulin secretion, a mixture of FFAs present in SBO was used, which enhanced GSIS similarly to digested SBO. After testing the individual acids present in SBO at specific concentrations, it was also confirmed that cPOA was responsible for the main stimulating effect, with its content in SBO being one of the highest of all identified FFAs (30 g/100 g oil)^24^. To accurately attribute the above-studied effects, control groups with other pure fatty acids present in SBO and/or key representatives of other bioactive lipids should be included, in order to assess their impact on lipid and glucose metabolism.

**Fig. 7:**
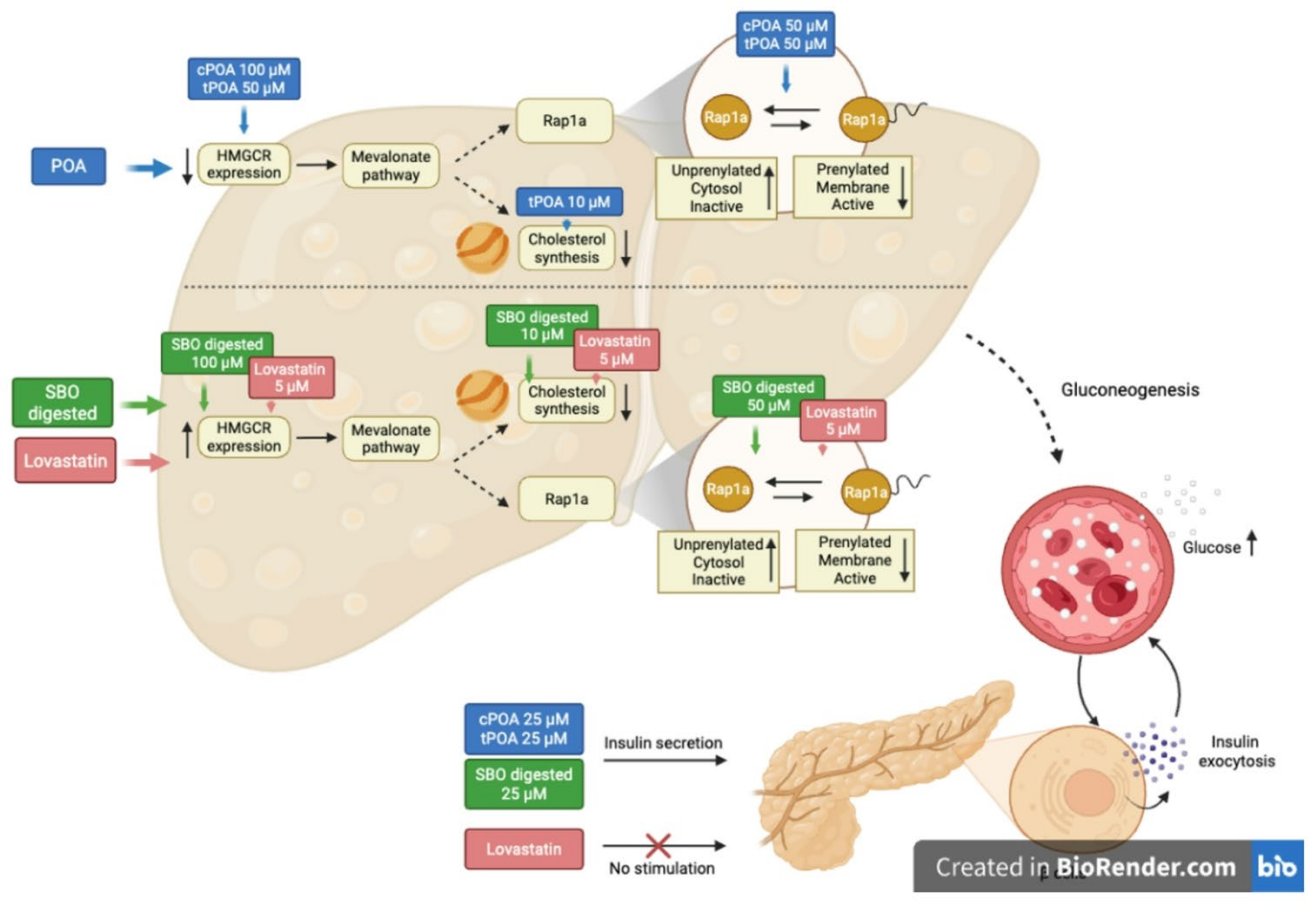
Effects of POA isomers, digested sea buckthorn oil (SBO) and lovastatin on cholesterol synthesis, Rap1a activation, and insulin secretion. The left panel outlines the pathway from HMG-CoA reductase (HMGCR) expression through the mevalonate pathway to cholesterol synthesis and subsequent Rap1a activation in liver cells. The right panel details how different concentrations of cPOA, tPOA, SBO, and Lovastatin influence the prenylation status and subcellular localization of Rap1a (cytosolic inactive vs. membrane-bound active form). The bottom section connects these molecular events to physiological outcomes, showing the impact on hepatic gluconeogenesis and pancreatic insulin secretion (created by BioRender.com).

These findings provide novel mechanistic insights and suggest that POA isomers and SBO may offer dual benefits in managing dyslipidemia and glucose intolerance without the adverse effects associated with statin therapy (Fig. 7).

## Conclusion

In summary, this study reveals that neither cPOA, tPOA, SBO, nor digested SBO diminishes the viability of HepG2 cells, even under steatotic conditions. By employing both normal and steatotic HepG2 cell models, we conducted a comparative analysis of the biological activities of these compounds for the first time. Our findings reveal their distinct effects on lipid accumulation, cholesterol levels, and GSIS.

Notably, tPOA treatment resulted in a significant reduction in TAG accumulation. Furthermore, low concentrations of cPOA, tPOA, SBO, and digested SBO triggered a reduction in cholesterol levels in steatotic cells, resembling the effect of lovastatin. Under steatotic conditions, both POA isomers, particularly at concentrations of 50 and 100 µM, demonstrated increased activity, elevating cytosolic Rap1 levels while altering its subcellular localization. Importantly, unlike lovastatin, which inhibited GSIS, both POA isomers and digested SBO significantly enhanced insulin secretion. These findings suggest that POA isomers and SBO may offer dual metabolic benefits: regulating lipid and cholesterol metabolism while supporting glucose homeostasis. Overall, this study highlights the therapeutic potential of palmitoleic acid isomers and sea buckthorn oil in managing metabolic disorders. However, further research is required to validate these findings and to elucidate the underlying molecular mechanisms, paving the way for potential clinical applications.

## Materials and methods

### Chemicals and reagents

RPMI Medium 1640 + GlutaMAX™, DMEM 4.5 g/l glucose, Phosphate Buffer Saline tablets and trypsin was from Gibco by Life Technologies (Carlsbad, CA, USA). Fetal Bovine Serum (FBS) Heat Inactivated, GeneMatrix Universal RNA Purification kit (with DNAse treatment), NG dART RT kit, and SG qPCR Master Mix were purchased from EURx Sp. z o. o. (Gdansk, Poland). Halt™ Protease Inhibitor Cocktail (100X), Mem-PER™ Plus Membrane Protein Extraction Kit, and PrestoBlue™ Cell Viability Reagent were purchased from Thermo Fisher Scientific Inc. (Waltham, MA, USA). Bio-Rad Protein Assay Dye Reagent Concentrate (Bradford Protein Assay) and Clarity™ Western ECL Substrate were from Bio-Rad Laboratories, Inc. (Hercules, CA, USA). Bovine Serum Albumin (BSA), Tween-20, Nonidet P-40 Substitute, Bovine Serum Albumin (BSA), palmitic acid (PA), oleic acid (OA), β-mercaptoethanol, NaCl, HEPES, KCl, CaCl_2_:2H_2_O, MgCl_2_:6H_2_O, Nile Red dye, lovastatin, Cholesterol Quantification Assay kit were from Sigma-Aldrich (St. Louis, MO, USA). Anti-Rap1A + Rap1B antibody (ab181858, 1:50,000 dilution) was from Abcam (Cambridge, UK). Antibody against β-actin (8457, 1:10,000 dilution) was purchased from Cell Signaling Technology, Inc. (Danvers, MA, USA). The secondary antibody used was Peroxidase AffiniPure™ Goat Anti-Rabbit IgG (H + L) (111-035-003, 1:50,000 dilution) from Jackson ImmunoResearch Laboratories, Inc. (West Grove, PA, USA). 0.45µm nitrocellulose membrane was purchased from Carl Roth GmbH + Co. KG (Karlsruhe, Germany). *Cis*-palmitoleic acid ((9Z)-hexadecenoic acid, C16:1(9Z), *cis* C16:1 n-7, cPOA) and *trans*-palmitoleic acid ((9E)-hexadecenoic acid, C16:1(9E), *trans* C16:1 n-7, tPOA) and Triglyceride Colorimetric Assay kit were purchased from Cayman Chemicals Company (Ann Arbor, MI, USA). Primers: βAKT (F: 5’-CTCGCCTTTGCCGATCC-3’ R: 5’-TCTCCATGTCGTCCCAGTTG-3’), HMGCR (F: 5’-GCAGGACCCCTTTGCTTAGA-3’ R: 5’- CCCTGACCTGGACTGGAAAC-3’) were purchased from Genomed S.A. (Warsaw, Poland). Isopropanol and chloroform were from ChemPur (Piekary Śląskie, Poland). All other chemicals were purchased from either Sigma-Aldrich or Thermo Fisher Scientific, Inc.

### Sea Buckthorn oil extraction and in vitro digestion

Sea Buckthorn Oil (SBO) was extracted from the pulp of berries using hexane in a Soxhlet apparatus as described previously^24^. Briefly, the fruits were cleaned, frozen, and placed in liquid nitrogen. They were then manually crushed and freeze-dried using an Alpha 2–4 LSC BASIC freeze-dryer (Martin Christ, Osterode am Harz, Germany). The seeds were manually extracted from the resulting dry mass of the whole fruits. The dried flesh of the sea buckthorn was prepared for the extraction of oil using hexane in a Soxhlet procedure, following the guidelines of PN-EN ISO 659:2010. The extracted oil was then subjected to in vitro digestion. 1 g of the oil was combined with 10 ml of a bile salt solution in PBS (0.188 g/10 ml), 2 ml of CaCl_2_ in water (0.551 g/20 ml), and 47.4 ml of NaCl in PBS (1.338 g/100 ml). The pH of the resulting solution was adjusted to 8.5 by adding either a 0.1 NaOH or a 0.1 HCl solution. The mixture was then homogenized using a T25 Ultra Turrax (IKA, Werke, Germany) and hydrolyzed in a 907 Titrando titrating device (Metrohm, Herisau, Switzerland). This process involved the use of 3 ml of pancreatic lipase in a PBS buffer (0.3 g of the enzyme in 5 ml, pH = 8.5) and 0.1 M water solutions of NaOH or HCl to stabilize the pH at 8.5. The hydrolysis process lasted for 3 h. Finally, the digested samples were dissolved in a PBS buffer until they reached a volume of 50 ml and stored in a frozen state.

### HepG2 cell culture and growth condition

The human hepatoma HepG2 cell line was purchased from the Leibniz Institute DSMZ—German Collection of Microorganisms and Cell Cultures (Leibniz, Germany). The cells were cultured in RPMI 1640 media supplemented with 10% fetal bovine serum (FBS) and antibiotics (100 U/mL penicillin, 50 µg/mL neomycin). The cells were maintained in a flask at 37 °C and 5% CO_2_. Steatosis conditions were induced by a mixed solution of oleic acid/palmitic acid (OA/PA) at concentrations of 300 µM of PA and 300 µM of OA. Following 24 h of exposure, HepG2 cells were evaluated microscopically and subjected to further analysis. Cells were incubated for 24 h, 48 h, or 72 h with POA isomers or crude and digested sea buckthorn oil at a final concentration of 10 µM, 25 µM, 50 µM or 100 µM (according to POA content).

### MIN6 cell culture and growth condition

The mouse insulinoma MIN6 cells were kindly provided by Prof. Peter Bergsten from Uppsala University (Sweden) with permission from Dr. Jun-ichi Miyazaki from Osaka University (Japan)^50^. The cells were grown in DMEM medium with 25 mM glucose, supplemented with 10% FBS, 50 µM β-mercaptoethanol and antibiotics (100 U/mL penicillin, 50 µg/mL neomycin). The cells were maintained at 37 °C with 5% CO_2_.

### Cell viability assay

HepG2 cells were seeded into 96-well plates at a density of 5 × 10^4^ cells per well in 100 µl of the culture medium. After 24-h of incubation, the culture medium was replaced with RPMI 1640 without FBS and supplemented with the compounds under investigation. The cells were then incubated for 24, 48, or 72 h. Cell viability was quantified using the PrestoBlue Cell Viability Reagent, following the manufacturer’s protocol. The fluorescent signal at F530/590 nm was measured using a Synergy 2 microplate reader (BioTek, Winooski, VT, USA). Cell viability was expressed as the percentage of compound-stimulated cell viability relative to untreated control cells.

### Intracellular lipids visualization via fluorescence nile red dye

Intracellular lipids were visualized using the fluorescence Nile Red dye. HepG2 cells were seeded into 96-well plates at a density of 1 × 10^5^ cells per well in 100 µl of the culture medium. Cells were incubated to achieve full confluence. After 24-h incubation with tested compounds cells were washed with PBS and fixed in 5% formaldehyde for 30 min. Cells were then washed again with PBS and stained with a Nile Red probe (1 µg/mL in PBS) for 20 min. Fluorescence intensity was measured at F485/530 nm. Microscopic observations were carried out on a contrast-phase and fluorescent microscope, Nikon TS100 Eclipse (Nikon, Tokyo, Japan) under x 100 magnification.

### Determination of triacylglycerols concentration

Total TAG concentrations were measured using a Triglyceride Colorimetric Assay Kit. HepG2 cells were seeded into Petri dishes (35 × 10 mm) at a density of 4 × 10^6^ cells in 5 ml of the culture medium. After 24-h incubation with tested compounds cells were rinsed with PBS and total cell lysates were prepared according to manufacturer’s protocol. Briefly, all reagents are diluted to achieve working solutions. Cells were collected with the use of a rubber policeman and suspended in Standard Diluent. Then cells were sonicated twenty times in one-second bursts. Subsequently, cells were centrifuged and TAG content was determined in supernatant with an enzyme cocktail prepared previously. TAG concentration was calculated based on the standard curve. The total protein concentration was determined via Bradford Protein Assay. Total TAG concentration was calculated and compared to total protein content (mg TAG/mg protein).

### Determination of cholesterol concentration

Total cholesterol content was measured with the use of a Cholesterol Quantification Assay kit. HepG2 cells were seeded into 6-well plates at a density of 1.5 × 10^6^ cells in 3 ml of the culture medium. After 24-h incubation with tested compounds cells were rinsed with PBS and total cell lysates were prepared according to manufacturer’s protocol. Briefly, all reagents are equilibrated to room temperature and diluted to achieve working solutions. Cells were extracted with the use of chloroform: isopropanol: Nonidet P40 (7:11:0.1) mixture. Samples then were centrifuged, supernatant collected and air dried at 50 °C. Dried lipids were dissolved in Assay Buffer. Cholesterol content was determined with an enzyme cocktail prepared previously. Finally, fluorescence intensity was measured (F535/587 nm) and cholesterol content was calculated based on the standard curve. The total protein concentration was determined via Bradford Protein Assay. Total cholesterol content was calculated and compared to the total protein content in the sample (mmol cholesterol/mg protein).

### RNA isolation, cDNA synthesis, and real-time RT-PCR

HepG2 cells were seeded into 6-well plates at a density of 2 × 10^6^ cells per well in 3 ml of RPMI medium. After 24-h incubation with tested compounds, the culture medium was removed and cells were rinsed with PBS buffer. RNA was extracted and purified with a GeneMatrix Universal RNA Purification Kit protocol. During the procedure samples were DNAse I treated (2 U/sample, 15 min). The concentration of RNA was measured spectrophotometrically. Next, RNA samples were reverse transcribed into cDNA with the use of an NG dART RT kit. Subsequently, RT-qPCR was performed using the SG qPCR Master Mix. The reaction was carried out on a CFX96 Touch Real-Time PCR Detection System (BioRad, Hercules, CA, USA). The sequences of specific primers were designed using the NCBI’s Primer-BLAST software (National Center for Biotechnology Information) based on receptors’ sequences from the GenBank database. The constitutively expressed βAKT gene was selected as an endogenous control to correct potential variation in RNA loading. The levels of HMGCR expression were normalized to the expressions of the housekeeping gene and control unstimulated cells according to Livak’s method. (2^−ΔΔCt^ )^51^.

### Western blot analyses (determination of Inhibition of Rap1a prenylation)

HepG2 cells were seeded into a 6-well culture plate at a density of 0.6 × 10^6^ cells per well in 2 ml of complete culture medium. The day after, a 1.2 ml serum-free culture medium was added and supplemented with investigated compounds and lovastatin. After 48-h of incubation, cells were rinsed with PBS and detached using a trypsin-EDTA solution. The cytosolic and membrane-rich protein fractions, containing Halt™ Protease Inhibitor Cocktail, were isolated from cell pellets using Mem-PER™ Plus Membrane Protein Extraction Kit according to the manufacturer’s instructions. The protein concentration in both fractions was measured by the Bradford Protein Assay. Cytosolic and membrane proteins in equal amounts (30 µg) were resolved by 12% SDS-PAGE gels and transferred to 0.45 μm nitrocellulose membrane. Membranes were blocked with 5% BSA in PBST (PBS supplemented with 0,1% Tween-20) at room temperature for 2 h. They were then incubated with β-actin or Rap1A/Rap1B antibodies for another 2 h, washed with PBST, and incubated with a secondary HRP-conjugated anti-rabbit antibody for 1 h at room temperature. Detection was followed by electrochemiluminescence (ECL) assay. Visualization of protein bands was performed using ChemiDocTM MP Imaging System (Bio-Rad, Hercules, CA, USA).

### In Silico molecular modeling

The structure of HMG-CoA reductase was obtained from the Protein Data Bank (ID: 1DQ9). The spatial structures of cPOA, tPOA, and lovastatin acid (LOV Acid) were downloaded from the PubChem database in sdf. format. Molecular docking was performed using ICM-Pro v.3.9-4a software (Molsoft L.L.C., San Diego, CA, USA, URL: https://www.molsoft.com/icmpro) into an adjusted catalytic center based on the localization of natural HMGCR ligand, HMG-CoA, from the crystal structure. Each ligand was docked to the protein ten times, with the thoroughness level set at 5. As a result, the structures, estimated energy, and the total docking score were obtained. All images were also presented with the ICM-Pro built-in display module.

### Glucose-Stimulated insulin secretion (GSIS)

MIN6 cells were seeded into a 24-well plate (2 × 10^5^ cells per well) in 1 ml of the culture medium. After 48 h, confluent cells were incubated at 37 °C for 60 min in calcium buffer Ca5 (125 mM NaCl, 25 mM HEPES, 6 mM KCl, 1.3 mM CaCl_2_:2H_2_O, 1.2 mM MgCl_2_:6H_2_O) supplemented with 2 mM glucose. Subsequently, cells were incubated in the new portion of the same Ca5-2 mM glucose buffer for 30 min with tested compounds applied at 10 or 25 µM concentration. Next, buffer samples were collected and the same cells were incubated for another 30 min in the Ca5 buffer supplemented with 20 mM glucose and tested samples. The buffer supernatants were collected again. Cells were lysed with 0.1 M HCl and cell lysates were used to assess the amount of total protein using Bradford Protein Assay. Collected buffer samples were subjected to insulin secretion measurements via competitive enzyme-linked immunosorbent assay (ELISA) (18).

### Statistical analysis

The results are presented as means of 3–6 repeated experiments (3–6 biological repeats each) ± SD. The data groups passed normality distribution tests and were compared using one-way Analysis of Variance (ANOVA) with the Bonferroni post hoc test. *p* < 0.05 was considered statistically significant. The results obtained after stimulation of the cell model with investigated compounds were compared to the respective solvents. GraphPad Prism v. 8.3 (GraphPad Software, La Jolla, CA, USA) was used to assess the statistical significance of the obtained data.

## Supplementary Information

Below is the link to the electronic supplementary material.

Supplementary Material 1

Supplementary Material 2

## Data Availability

All relevant data for this study is included in this manuscript. The data which were not shown are available upon request from the corresponding author.

## Abbrevations

cPOA: *cis*-palmitoleic acid
CYP7A1: cholesterol 7-alpha hydroxylase
EPA: eicosapentaenoic acid
FFA: eicosapentaenoic acid
GGPP: geranylgeranyl pyrophosphate
GGT-I: geranylgeranyltransferase type I
GSIS: glucose stimulated insulin secretion
HDL: high-density lipoprotein
HFD: high-fat diet
HMG-CoA: HMG-CoA
HMG-CoA: 3-hydroxy-3-methylglutaryl coenzyme A reductase
LDL: low-density lipoprotein
MASLD: metabolic dysfunction-associated steatotic liver disease
OA: oleic acid
PA: palmitic acid
PCSK-9: proprotein convertase subtilisin-kexin type 9
POA: palmitoleic acid
PTM: post-translational modification
PUFA: polyunsaturated fatty acid
SBO: sea buckthorn oil
Sp1: specific protein 1
SREBP-2: steroid response element binding protein-2
TAG: triglyceride
TBA: total bile acid
tPOA: trans-palmitoleic acid
UFA: unsaturated fatty acid
