# Supplementary material for: Comparative study of palmitoleic acid, sea buckthorn oil, and lovastatin in hepatocellular steatosis model
Source: Sci Rep. 2026 Jan 24;16:6135. doi: 10.1038/s41598-026-37006-y (PMC12902076; doi:10.1038/s41598-026-37006-y)
Supplement: Supplementary file 1 — Supplementary Material 1 [file 41598_2026_37006_MOESM1_ESM.docx]

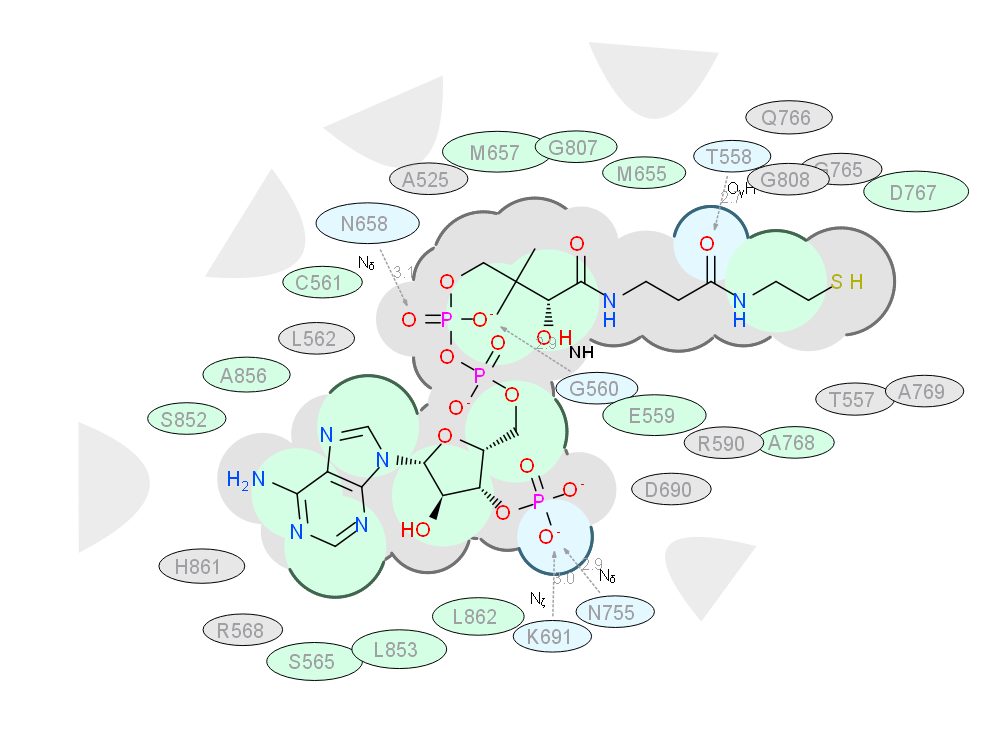

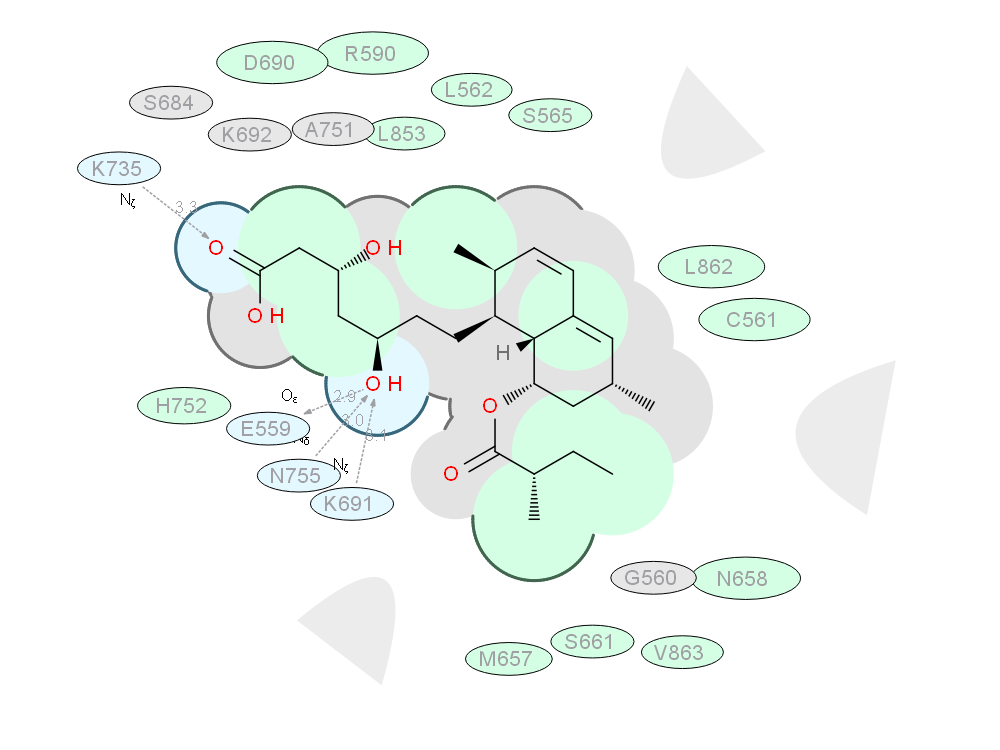

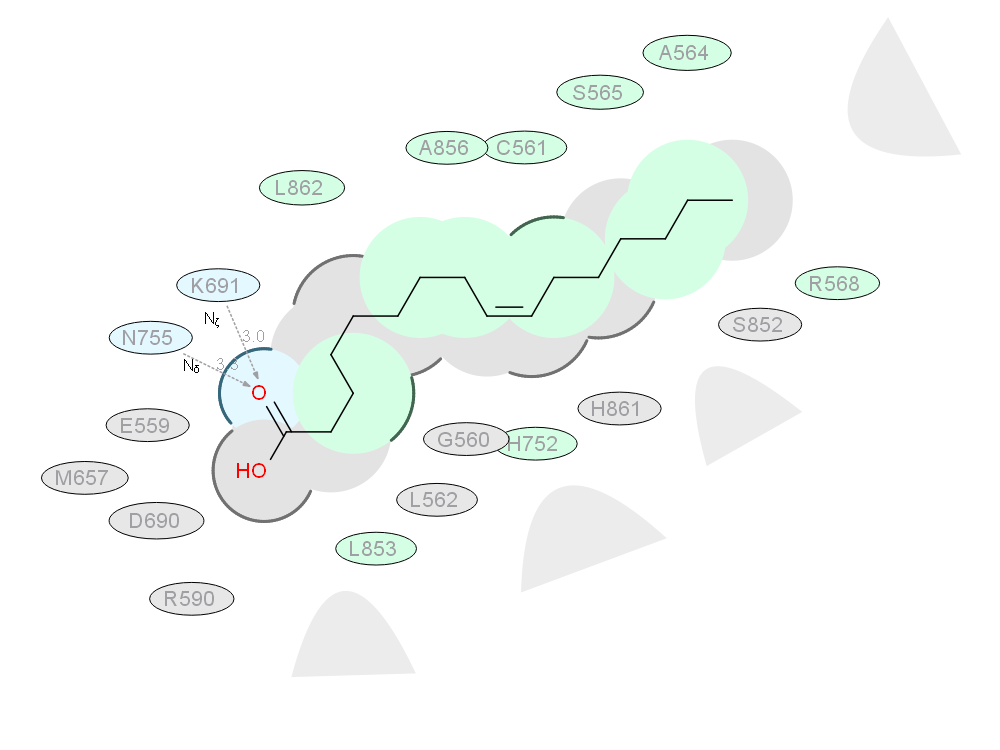

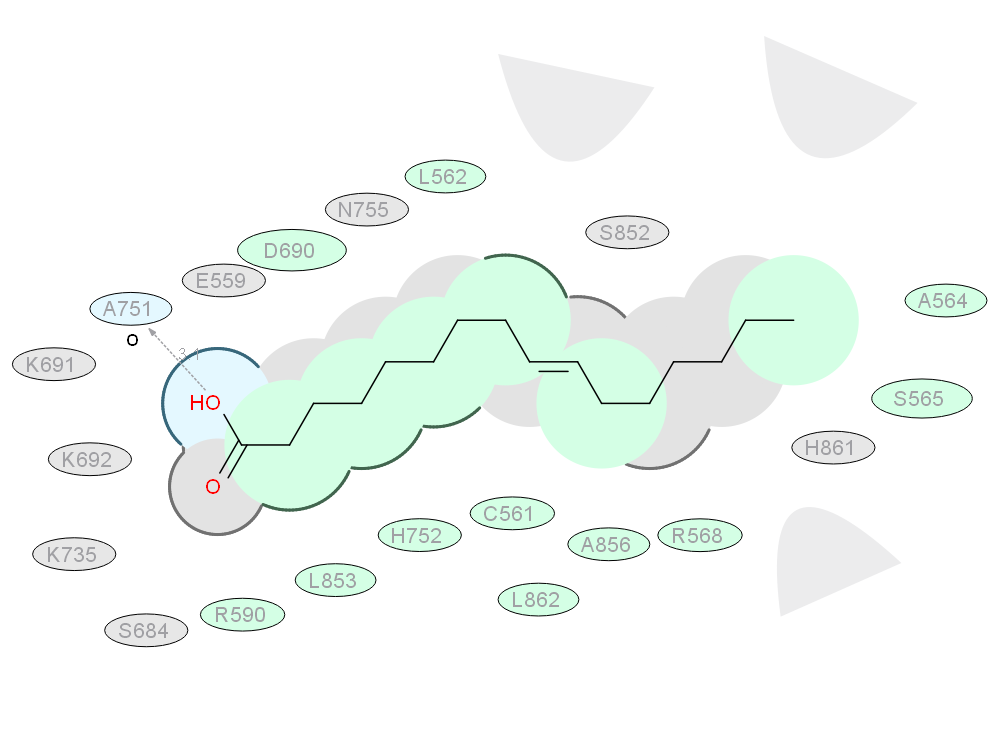


Key interactions after docking (2D diagram)

**HMG-CoA**

**LOV ACID**

**cPOA**

**tPOA**

**Figure S1**. The 2D interaction diagram, prepared using ICM-Pro, visualizes the binding mode of the ligand within the protein pocket. Hydrophobic regions of the binding site are highlighted in green, indicating non-polar areas. Blue shaded regions represent hydrogen bond acceptor potentials within the binding site. Hydrogen bonds between the ligand and amino acid residues are represented by dashed arrows. Grey parabolas illustrate the solvent-accessible surface around the ligand, emphasizing its exposure to the surrounding environment. The size of residue ellipses reflects the strength of their contacts with the ligand, and the proximity of labels correlates with physical closeness in three-dimensional space.
