# Supplementary material for: Comparative study of palmitoleic acid, sea buckthorn oil, and lovastatin in hepatocellular steatosis model
Source: Sci Rep. 2026 Jan 24;16:6135. doi: 10.1038/s41598-026-37006-y (PMC12902076; doi:10.1038/s41598-026-37006-y)

Supplementary information file

The supplement contains images of unprocessed membranes from Western blot analyses, where two different exposure times were used to visualize the edges of the membranes. The membranes were cut before antibody hybridization, and the blots show single bands.

Cell not treated with OA/PA mixure
Cytosol fraction

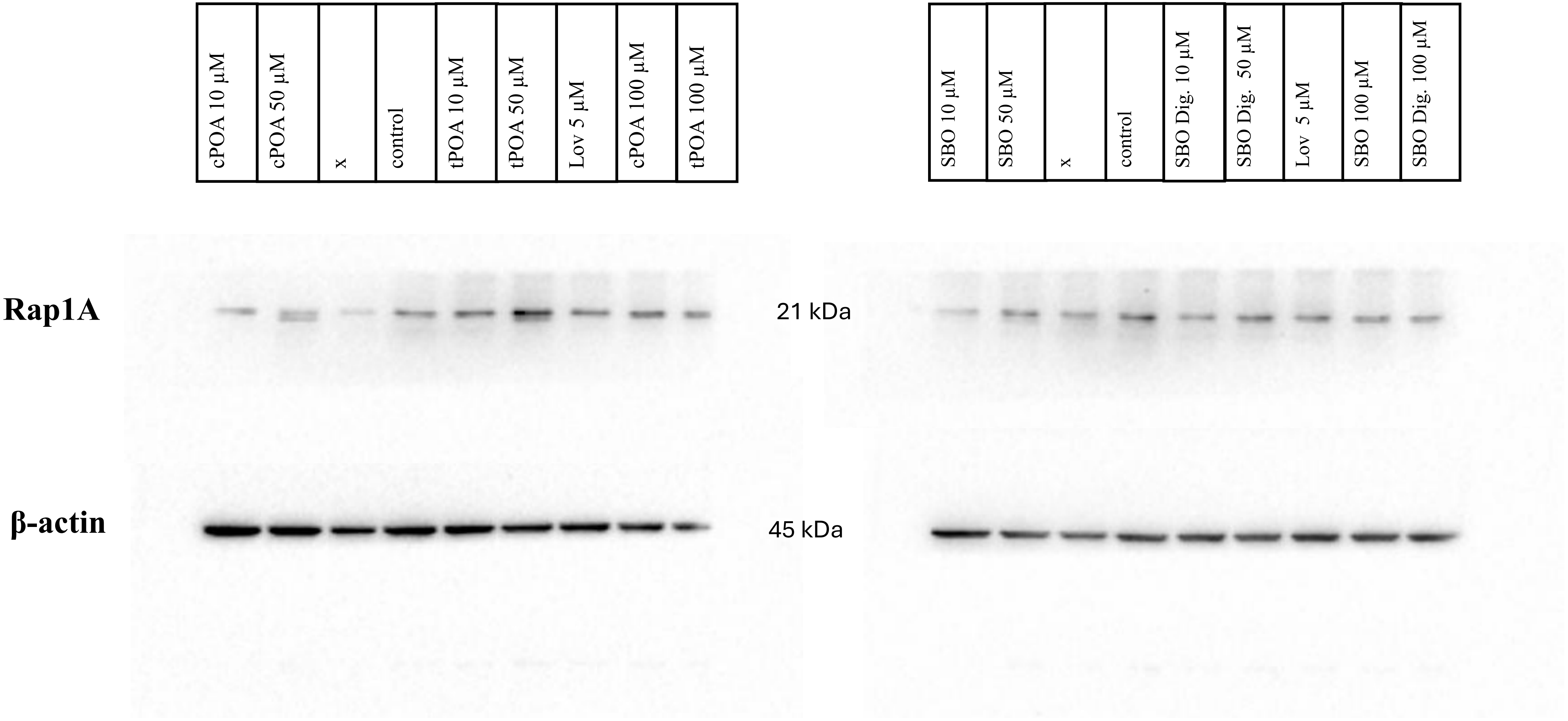

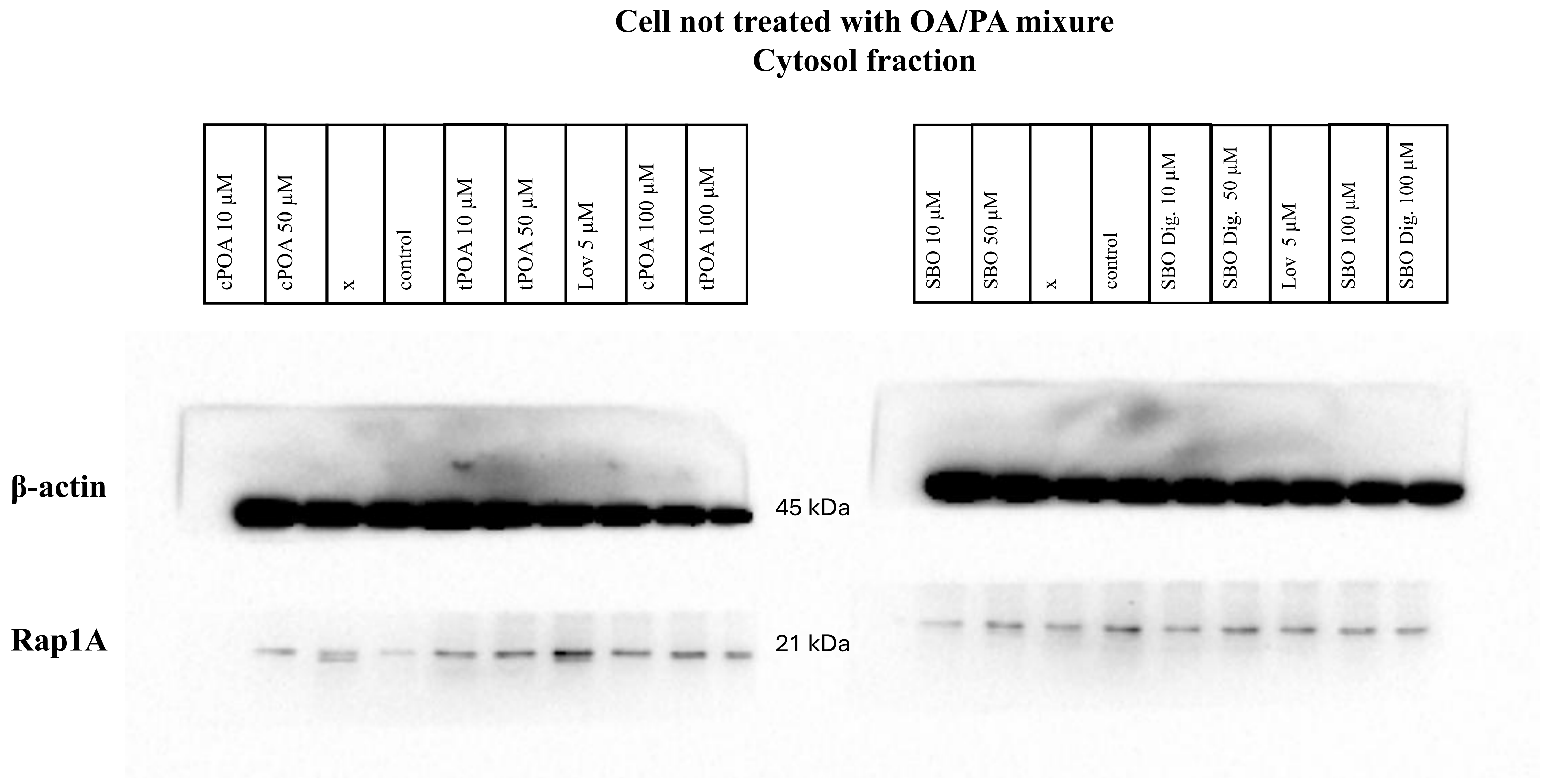
Cell not treated with OA/PA mixure
Cytosol fraction
cPOA 10 µM
cPOA 50 µM
x
control
tPOA 10 µM
tPOA 50 µM
Lov 5 µM
cPOA 100 µM
tPOA 100 µM
SBO 10 µM
SBO 50 µM
x
control
SBO Dig. 10 µM
SBO Dig. 50 µM
Lov 5 µM
SBO 100 µM
SBO Dig. 100 µM
β-actin
Rap1A
45 kDa
21 kDa

Cell not treated with OA/PA mixure
Membrane fraction

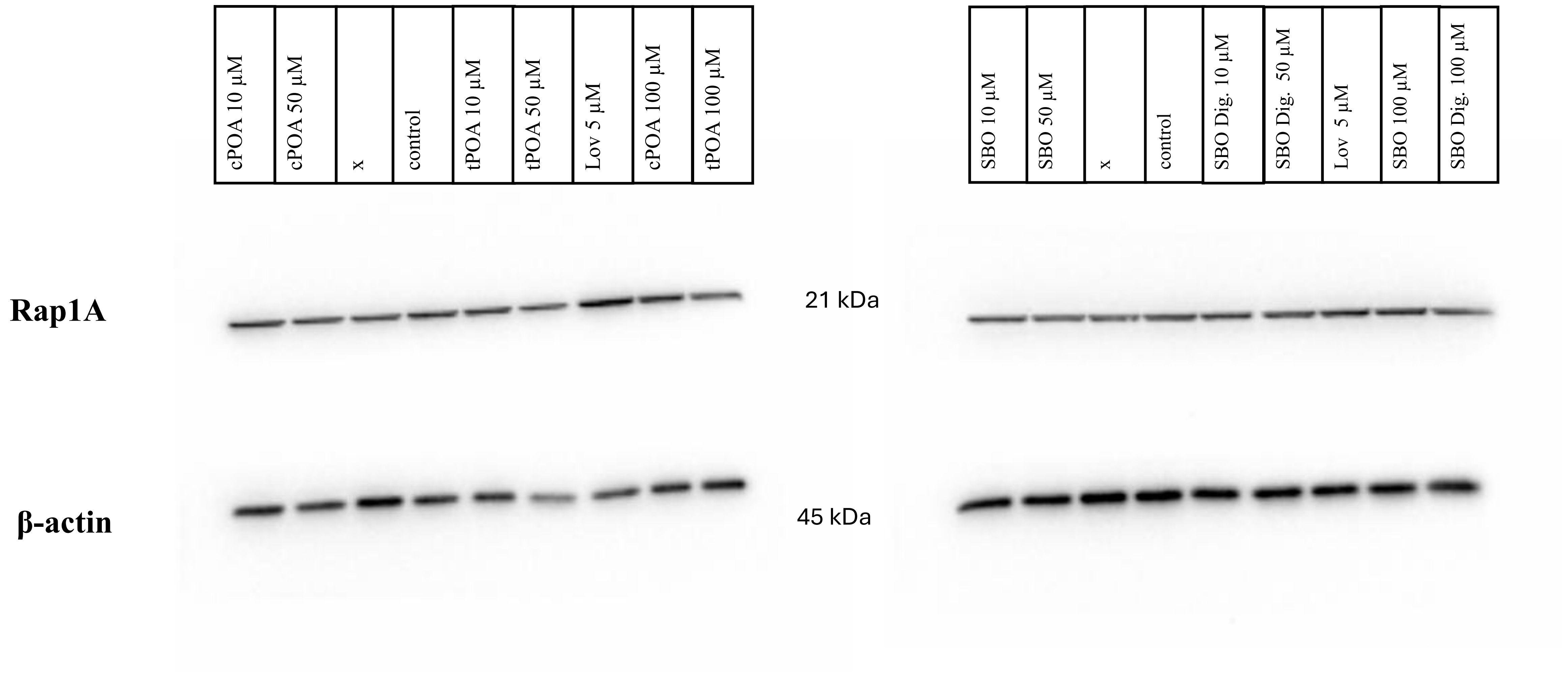

Cell not treated with OA/PA mixure
Membrane fraction

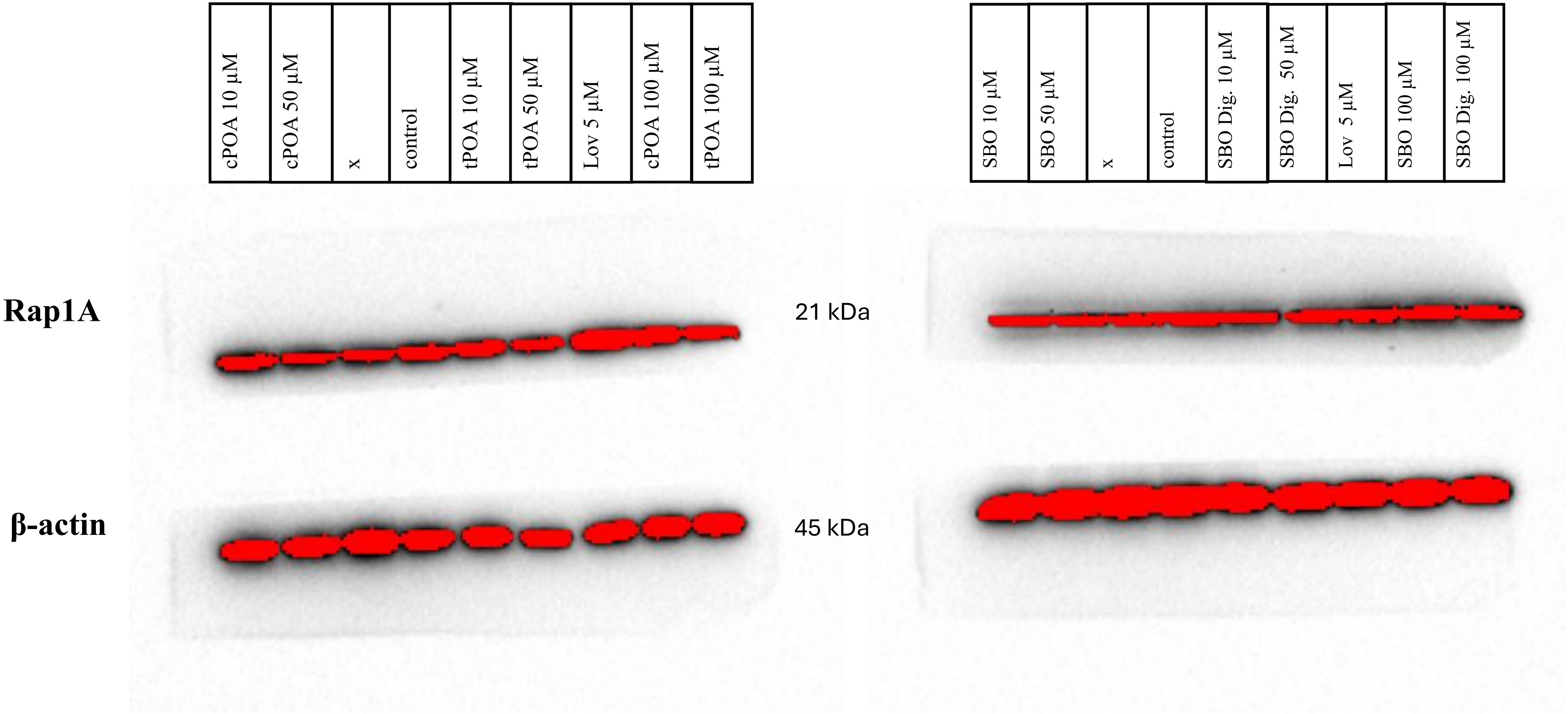

FFA-induced steatosis
Cytosol fraction

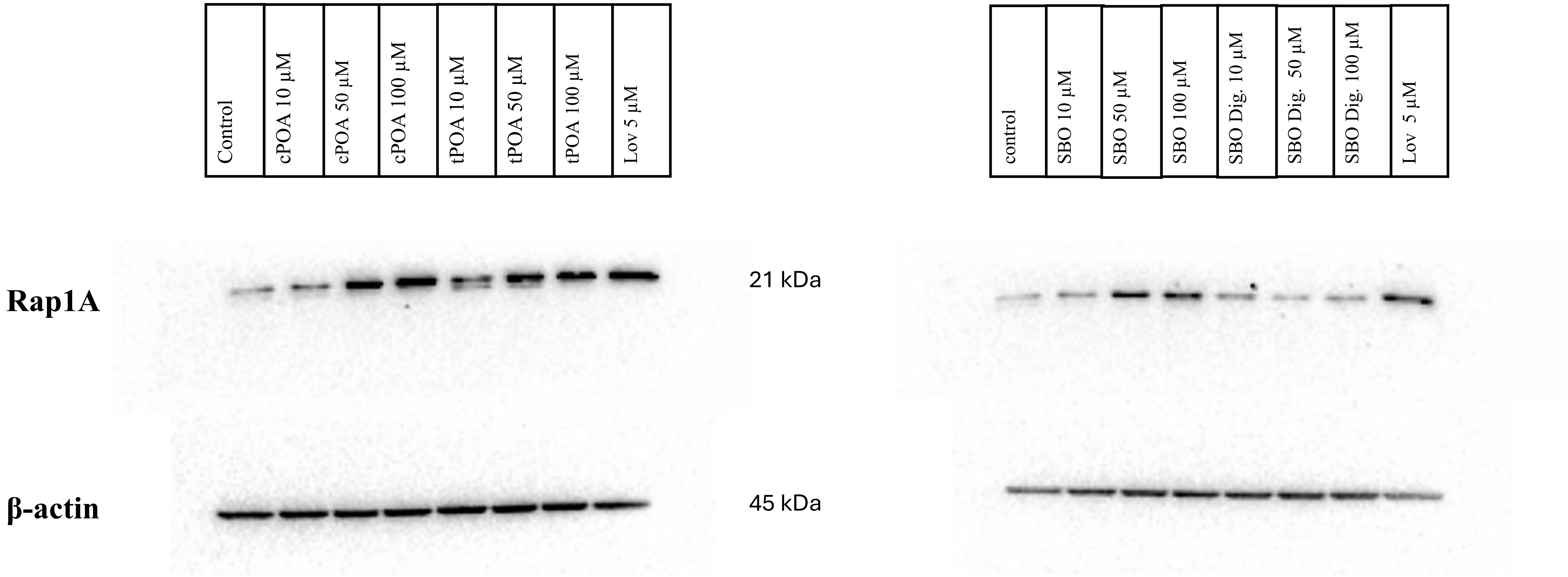

FFA-induced steatosis
Cytosol fraction

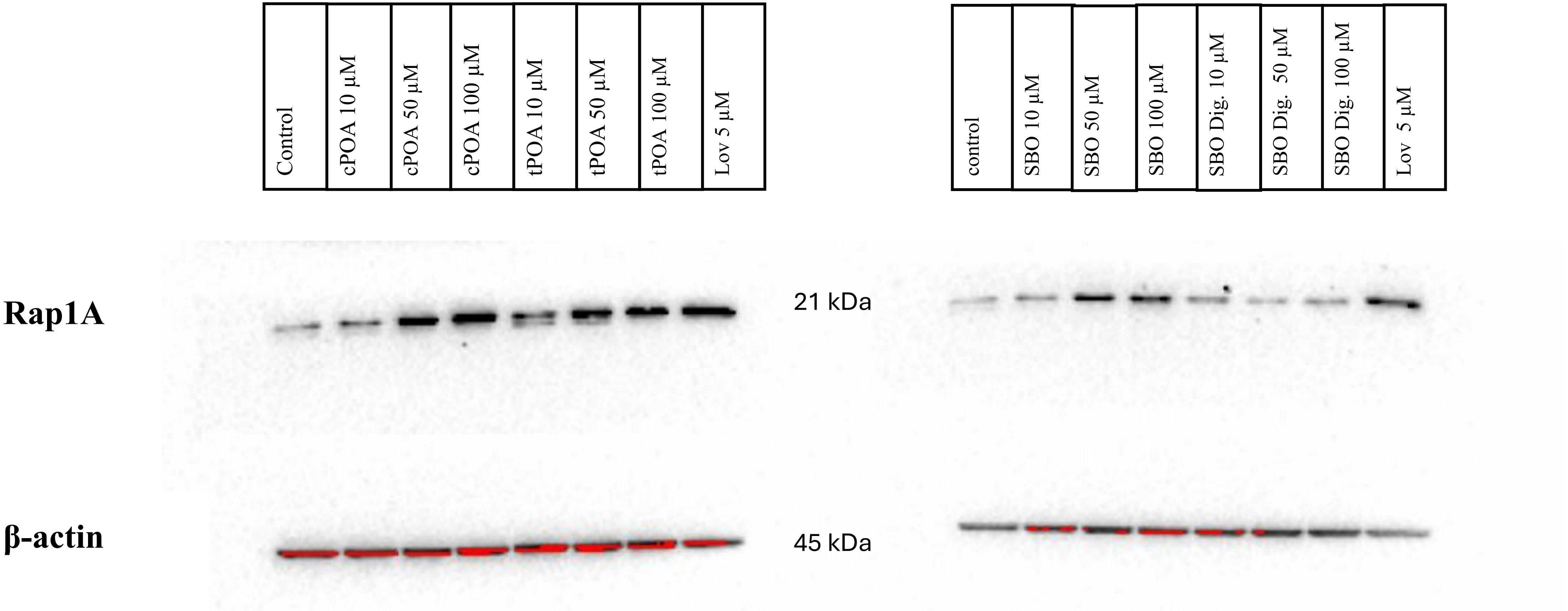

FFA-induced steatosis
Membran fraction

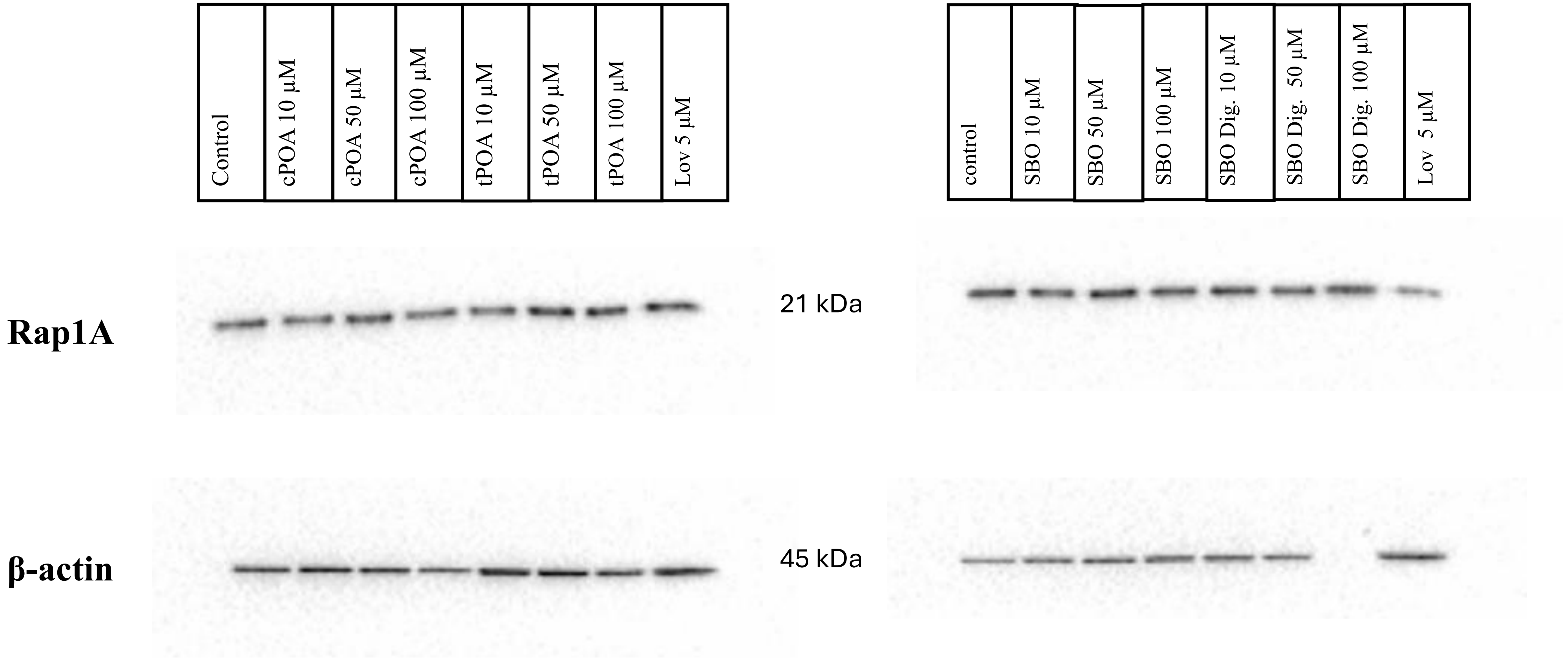

FFA-induced steatosis
Membran fraction

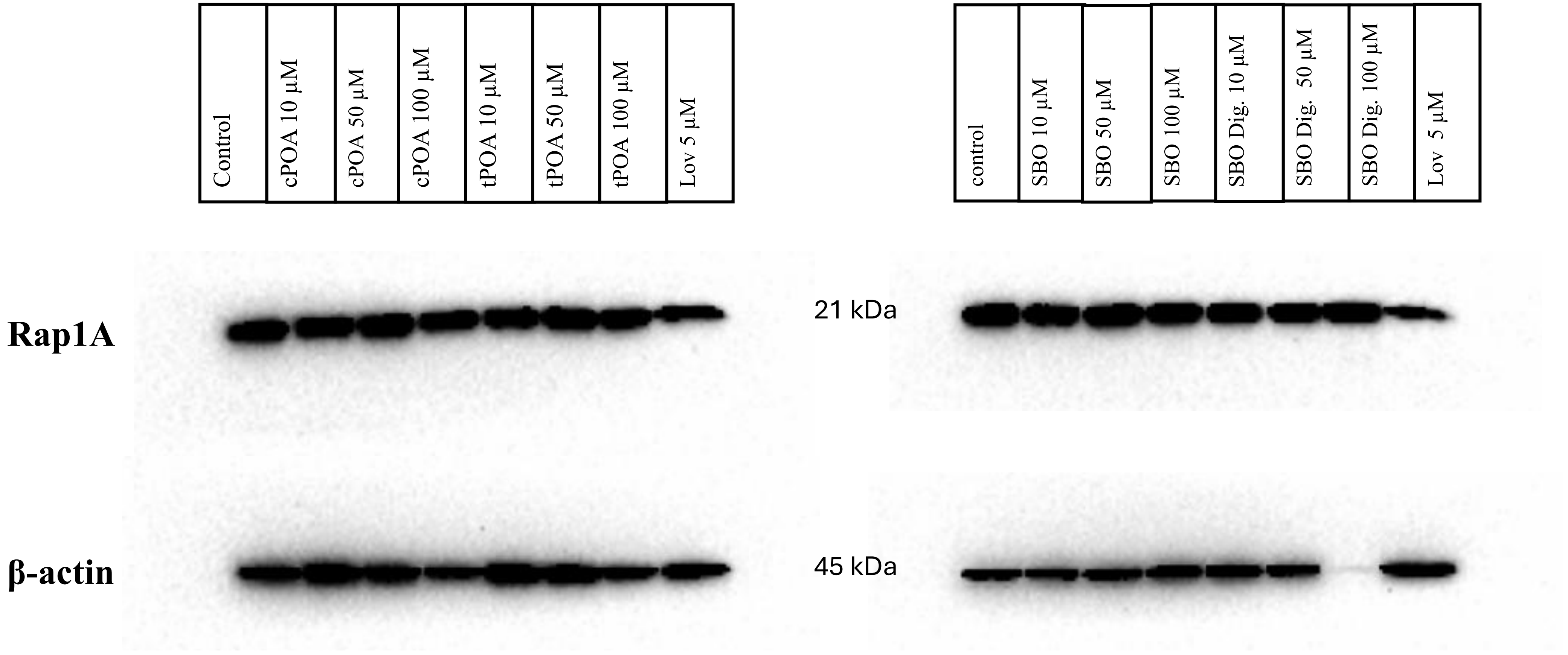

FFA-induced steatosis
Membran fraction

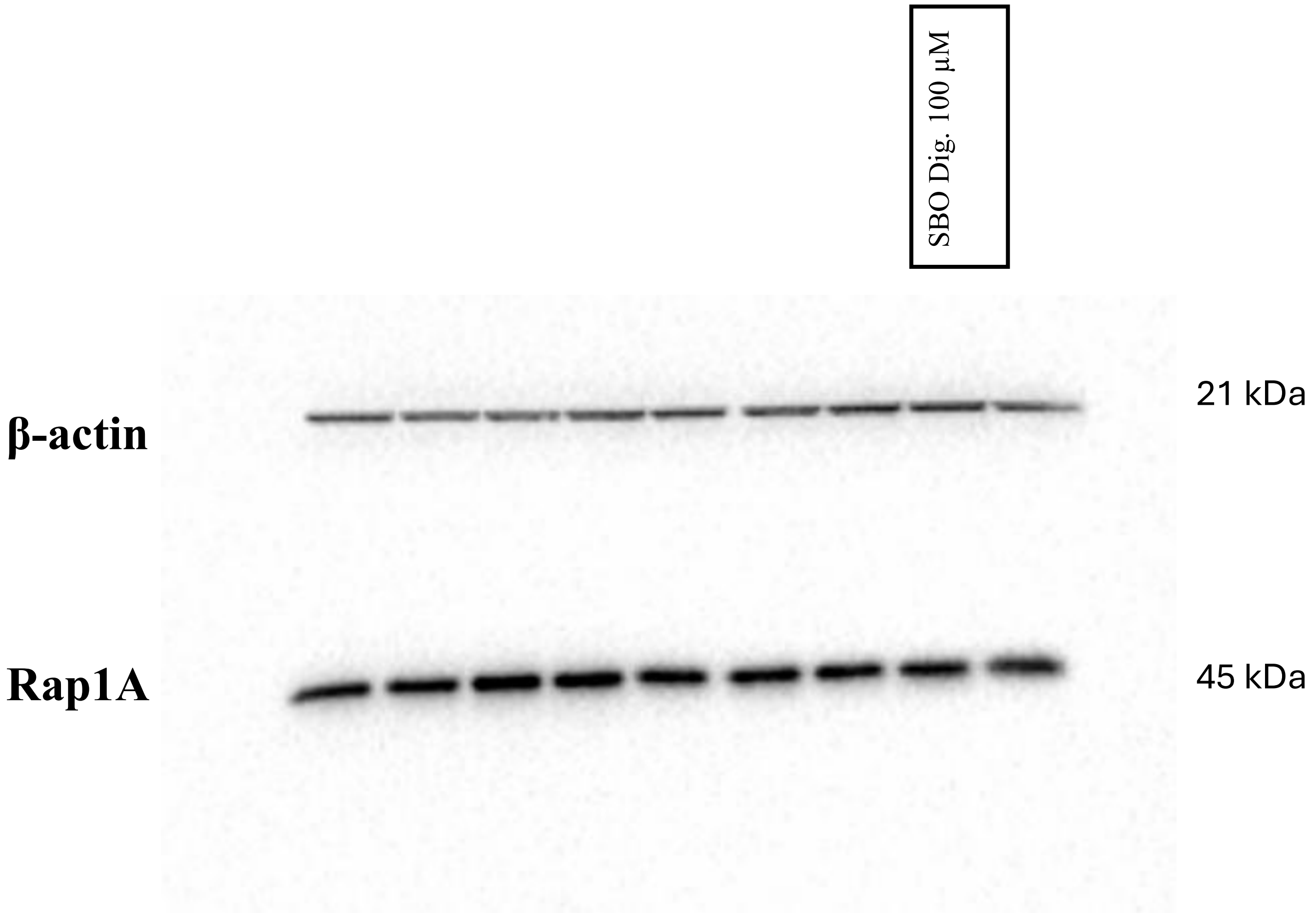

FFA-induced steatosis
Membran fraction

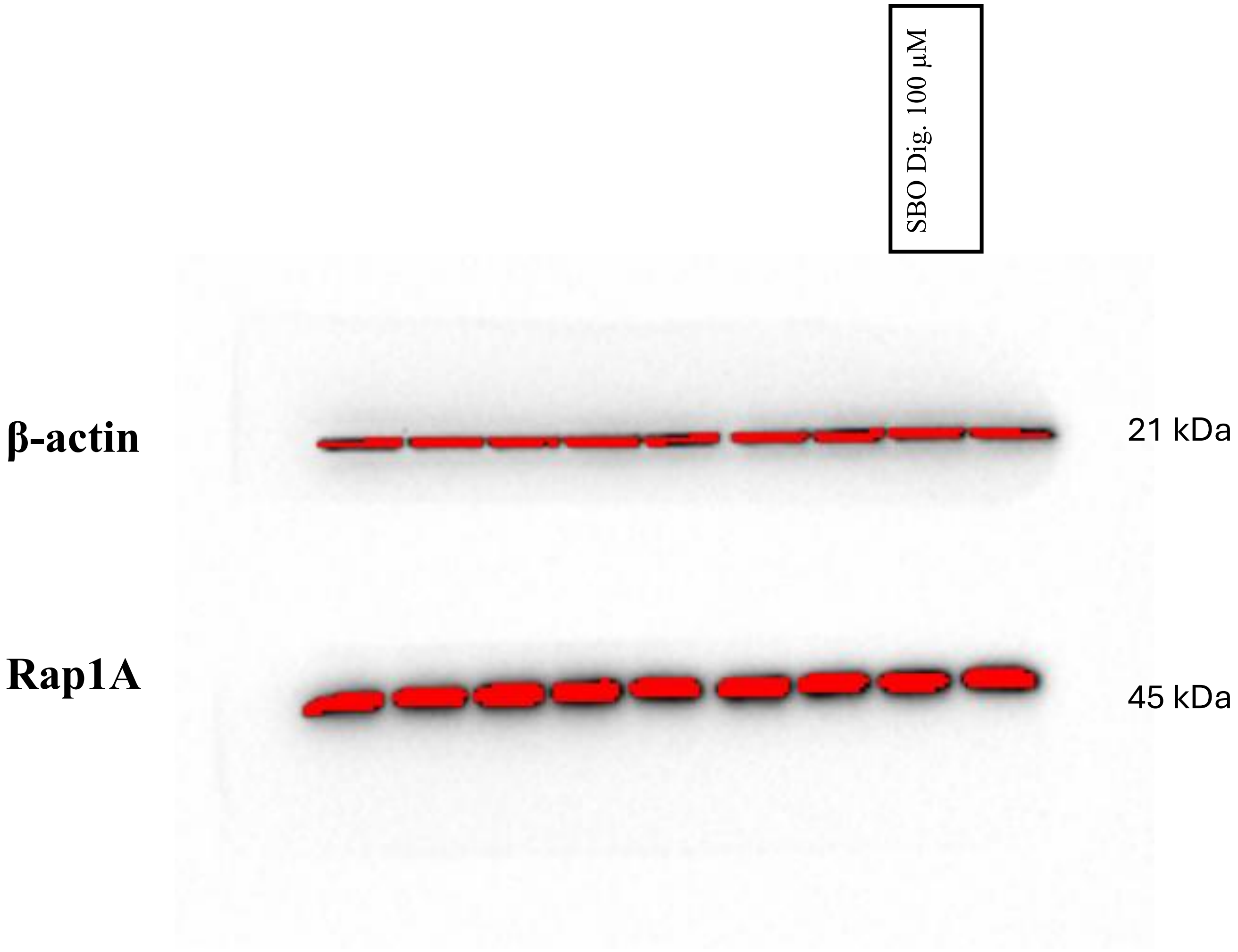

Supplement: Supplementary file 2 — Supplementary Material 2 [file 41598_2026_37006_MOESM2_ESM.pdf]
